# Putative Signals of Generalist Plant Species Adaptation to Local Pollinator Communities and Abiotic Factors

**DOI:** 10.1093/molbev/msad036

**Published:** 2023-02-16

**Authors:** Léa Frachon, Luca Arrigo, Quint Rusman, Lucy Poveda, Weihong Qi, Giovanni Scopece, Florian P Schiestl

**Affiliations:** Department of Systematic and Evolutionary Botany, University of Zurich, Zurich, Switzerland; Department of Systematic and Evolutionary Botany, University of Zurich, Zurich, Switzerland; Department of Systematic and Evolutionary Botany, University of Zurich, Zurich, Switzerland; Functional Genomics Center Zurich, ETH Zurich/University of Zurich, Zurich, Switzerland; Functional Genomics Center Zurich, ETH Zurich/University of Zurich, Zurich, Switzerland; SIB Swiss Institute of Bioinformatics, 1202 Geneva, Switzerland; Department of Biology, University of Naples Federico II, Complesso Universitario MSA, Naples, Italy; NBFC: National Biodiversity Future Center, Palermo 90133, Italy; Department of Systematic and Evolutionary Botany, University of Zurich, Zurich, Switzerland

**Keywords:** *Brassica incana*, generalist-pollinated plant species, local adaptation, natural populations, plant–pollinator interactions, genome-environmental association

## Abstract

The reproductive success of flowering plants with generalized pollination systems is influenced by interactions with a diverse pollinator community and abiotic factors. However, knowledge about the adaptative potential of plants to complex ecological networks and the underlying genetic mechanisms is still limited. Based on a pool-sequencing approach of 21 natural populations of *Brassica incana* in Southern Italy, we combined a genome-environmental association analysis with a genome scan for signals of population genomic differentiation to discover genetic variants associated with the ecological variation. We identified genomic regions putatively involved in the adaptation of *B. incana* to the identity of local pollinator functional categories and pollinator community composition. Interestingly, we observed several shared candidate genes associated with long-tongue bees, soil texture, and temperature variation. We established a genomic map of potential generalist flowering plant local adaptation to complex biotic interactions, and the importance of considering multiple environmental factors to describe the adaptive landscape of plant populations.

## Introduction

Many flowering plant species interact simultaneously with different functional groups of pollinators and are thus termed plants with generalized pollination systems (Albrecht et al. 2012). By being able to achieve pollination through an assemblage of generalist and specialist pollinators of various taxa, widely distributed generalist plant species (Waser et al. 1996; Johnson and Steiner 2000) appear robust to pollinator changes within mutualistic networks (Bascompte and Jordano 2007; Thébault and Fontaine 2010; Burkle et al. 2013; Zografou et al 2021). Although generalist plant species are keystones in mutualist interaction networks, we know little about the adaptive potential of these plants to variable pollinator communities. Only a handful of studies have demonstrated the role of pollinator assemblages on floral evolution in generalist plant species (Gòmez, Abdelaziz, et al. 2009; Sahli and Conner 2011; Gòmez et al. 2015; Sobral et al. 2015; Schiestl et al. 2018; de Manincor et al. 2021). For instance, it has recently been shown that pollinator communities can drive flower shape evolution in the genus *Erysimum* (Gòmez et al. 2015), or geographic variation in flower scent (de Manincor et al. 2021). However, floral evolution in generalist plant species appears to be complex (Gòmez et al. 2015), probably involving independent and genetically linked phenotypic traits associated with pollinator preferences (Frachon et al. 2021; Ohashi et al. 2021). To understand if and how generalist plant species adapt to their local pollinator communities, we need to investigate the underlying genomics. This will help us understand the coevolution of generalist plant–pollinator networks.

Plant–pollinator interactions are influenced by abiotic factors (Tylianakis et al. 2008; Chamberlain et al 2014; Antiqueira et al. 2020). For instance, climate change can induce mismatches between plants and pollinators due to nonsynchronized phenology shifts (Hegland et al. 2009; Petanidou et al. 2014), or changes in plant attractiveness to pollinators (Petanidou and Smets 1996; Hoover et al. 2012; Herrera and Medrano 2017; Descamps et al. 2021). Moreover, soil heterogeneity can strongly affect plant attractiveness to pollinators through changes in nectar secretion, production of pollen, and floral scent (Burkle and Irwin 2009; Majetica et al. 2017; David et al. 2019; Carvalheiro et al. 2021). Understanding how both pollinator communities and abiotic factors simultaneously drive the evolution of phenotypic traits and their associated genomic regions is a current challenge requiring a holistic approach from ecology to genomics (Clare et al. 2013; López-Goldar and Agrawal 2021).

Natural selection drives variation in phenotypic traits towards a local optimum by changes in allele frequencies of associated genomic regions. Genome-environment association (GEA) analysis is a powerful approach to identify those genomic regions involved in potential adaptive responses of organisms to complex combination of environmental factors without phenotypic characterization (de Mita et al. 2013; Günther and Coop 2013). This approach takes advantage of the genetic fingerprint left by selective pressures due to environmental variation among natural populations. By detecting genomic regions highly correlated with environmental variables and combined with methods testing for evidence of signatures of selection, GEA successfully detects the putative loci underlying local adaptation, that is, the genomic local adaptation (Hancock et al. 2011; Hoban et al. 2016; Sork 2018). Although commonly used to understand the genetic architecture of plants involved in responses to climate change (Hancock et al. 2011; Lasky et al. 2015; Pluess et al. 2016; Cortés and Blair 2018; Frachon et al. 2018), the GEA approach has recently shown its effectiveness in unraveling the genetic variants of *A. thaliana* associated with adaptative responses to complex biotic interactions such as plant—leaf microbiomes (Horton et al. 2014) and plant—plant communities (Frachon et al. 2019).

In our study, we adopted a GEA approach to understand the genomic regions involved in putative signals of local adaptation of the generalist-pollinated plant *Brassica incana* to its pollinator community (visitation by pollinator functional categories, and pollinator community composition) as well as to potential interacting effects with climatic and edaphic (soil composition and texture) variables. By characterizing 61 ecological factors, de novo assembly of the *B. incana* reference genome, and pool-sequencing of 21 natural populations of *B. incana* in Southern Italy for 5,530,708 single nucleotide polymorphisms (SNPs), we fine mapped QTLs associated with variation in pollinator communities, climate, and soil. This approach was combined with a genome scan for signatures of spatial genomic differentiation (X^T^X) and enrichment in SNPs with high genomic differentiation to detect putative signatures of selection. Altogether, we identified genomic regions involved in putative signals of adaptation of a plant species with a generalized pollination system to a complex ecological network and abiotic factors.

## Results

### Variation in Ecological Factors Among 21 Natural Populations of *Brassica incana*

**Fig. 1.**
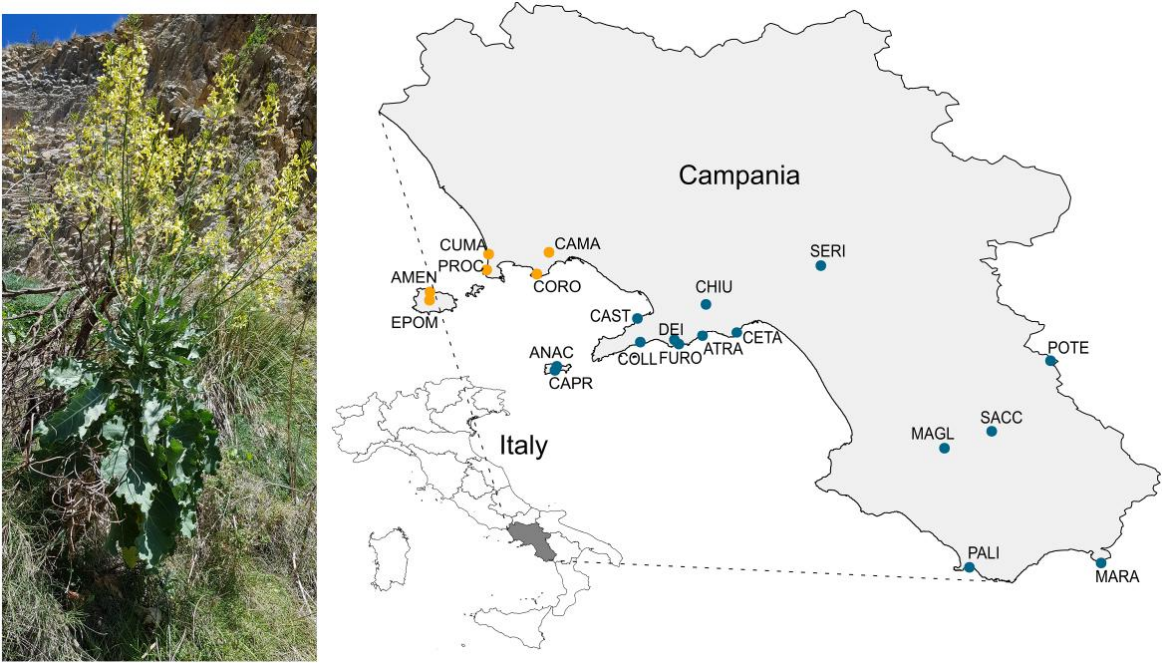
Distribution of *Brassica incana* natural populations. On the left is a photograph of a flowering *B. incana* in the PALI population. On the right, the Campania region is represented in dark gray on the Italy map. The 21 natural populations of *B. incana* are indicated with colored dots on the map. The orange dots indicate six populations on tuff soil and the blue dots 15 populations on limestone soil. See supplementary table S1, Supplementary Material online for full name of populations. Courtesy of Léa Frachon.

**Fig. 2.**
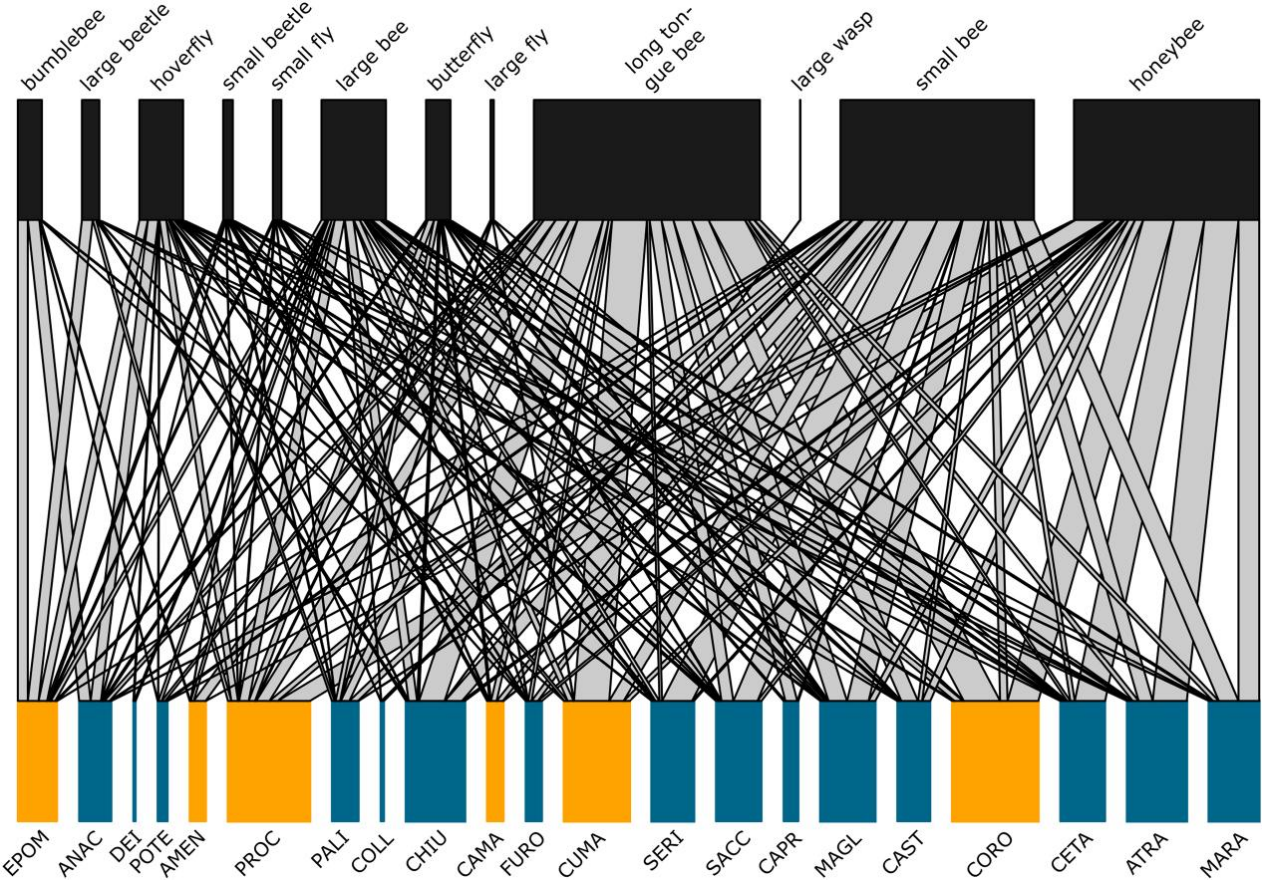
*Brassica incana*—pollinator interaction network for 21 natural populations. The upper part of the figure represents the 12 functional categories of pollinators. The size of boxes represents the total number of visits per functional category of pollinators observed in all 21 populations in springs of 2018 and 2019. The lower part of the figure represents the 21 natural populations of *B. incana* colored according to their soil type (tuff soil in orange, limestone soil in blue). The size of the boxes represents the total number of visits of all categories of pollinators combined per population. The width of the lines connecting functional categories of pollinators to populations indicates the proportion of visits observed per pollinator category within each population. Pollinator functional groups and plant population are ordered as such leading to as few crossings of interactions as possible.

Pollinator communities were characterized during the spring seasons of 2018 and 2019 by observing the visits of functional categories of pollinators to *B. incana* plants within 21 natural populations (fig. 1, supplementary table S1, Supplementary Material online). Flower visitors were grouped into 12 functional categories, that is, bumblebees, long-tongue bees, other large bees (called large bees), small bees, honeybees, large wasps, small flies, large flies, hoverflies, small beetles, large beetles, and butterflies (fig. 2). To characterize differences in pollinator communities among populations, we performed a plant–pollinator network analysis based on the total number of pollinator visits by functional categories. We graphically depicted pollinator community structure for the different *B. incana* populations in figure 2, where the number of visits by functional categories of pollinators is linked to the different plant populations. Pollinator communities were mainly dominated by long-tongue bees, small bees, honeybees, large bees, hoverflies, and bumblebees in decreasing order (fig. 2). Moreover, visitation by functional categories of pollinators varied among the 21 populations (fig. 2), leading to variation in pollinator community composition as estimated by the partner diversity index (minimum = 0.63, maximum 1.80, average = 1.28) (supplementary table S2, Supplementary Material online). The calculated indices from the network analysis (supplementary table S2, Supplementary Material online) suggest that *B. incana* plants in natural populations can be considered generalist. We found high normalized degree index values (i.e., the number of observed links between pollinator functional groups and a plant populations) showing a high number of realized *B. incana*—pollinators links among populations (minimum = 0.17, maximum = 0.83, average = 0.56, supplementary table S2, Supplementary Material online) and low *d*-index values, that is, the degree of specialization (0 = highly generalized, 1 = highly specialized) of each plant population (minimum = 0.04, maximum = 0.53, average = 0.17, supplementary table S2, Supplementary Material online).

Among the 21 natural populations, 28 of 61 characterized ecological variables were highly correlated (Spearman *ρ* > 0.8) and were all discarded from the genomic analyzes (supplementary fig. S1, Supplementary Material online). These highly correlated variables concerned mainly pollinator community indices (four out of eight variables) and climate variables (17 variables out of 20). While we had a clear differentiation of tuff *versus* limestone soils following the Northwest—Southeast axis among the 21 populations (due to the geography of Southern Italy), the different characteristics of these soils were not correlated with each other (supplementary fig. S1, Supplementary Material online). The principal component analysis showed that the six populations in tuff soil (AMEN, CAMA, CORO, CUMA, PROC, and EPOM) were ecologically similar and differentiated from other populations by the visits of hoverflies, the visits of large beetles, the mean annual precipitation, and the species strength (an index for pollinator community structure, supplementary fig. S2, Supplementary Material online). The visits of bumblebees, large bees, and *d*-index values (another index of pollinator community structure) were correlated to the fine sand, coarse silt, Fe, and summer precipitation in the ecological space created by the two first axis of the principal component analysis (supplementary fig. S2, Supplementary Material online).

### Annotated Reference Genome of *Brassica incana*

The final assembled sequences of the *Brassica incana* reference genome were organized into 1,339 contigs, which were scaffolded into 139 super-scaffolds using Bionano optical map (supplementary table S5, Supplementary Material online). The 139 super-scaffolds were used in our study, including a total sequence length of 617 Mbp, scaffold N50 of 12 Mbp, and a longest sequence at 32 Mbp, with a BUSCO completeness score of 97.7% (supplementary table S5, Supplementary Material online). Sequencing data from Pacbio and Illumina used for this study are available in the European nucleotide archive (ENA) database (accession number PRJEB54646). The bionano raw data and assembled optical maps are available at National Library for Biotechnology Information (NCBI) database (sample name PRJNA859008).

In total 51,001 genes were predicted, including 50,895 proteins (from the iprscan) divided into 1,112 different categories of Gene Ontology (GO) terms. In comparison, the reference genome of *Brassica oleracea* (genome size = 488.6 Mb) is composed of 53,125 genes, and that of *Arabidopsis thaliana* contains 38,311 genes (genome size = 119.1 Mb) in the NCBI database.

### Genomic Architecture Associated With Ecological Variation

After mapping the 21 pool-sequences from 21 natural populations to the generated *B. incana* reference genome, we estimated the allele frequencies across the 139 super-scaffolds for a final number of 5,530,708 SNPs. An important concern in GEA is the effect of population structure on association scores, which can increase the number of false positives. In our study, we attempted to reduce and correct this effect in multiple ways. First, we worked at a regional geographical scale which should reduce the effect of population structure (Bergelson and Roux 2010; Frachon et al. 2018, 2019). Using singular value decomposition (SVD) of the population variance-covariance matrix Ω, we observed a dispersion of populations in the genomic space represented by the first PC_genomic_ explaining 94.3% of genomic variance (supplementary fig. S3, Supplementary Material online). While we observed a geographic pattern along the Northeast—Southwest axis (linear model for PC1_genomic_; latitude: *t-value* = 3.24, *P* = 0.005, longitude: *t-value* = 3.31, *P* = 0.004, latitude*longitude: *t-value* = −3.33, *P* = 0.004, adjusted *R*^2^ = 47.1%), the variation of most ecological factors was weakly (nonsignificant) correlated with the genomic variation (supplementary table S6, Supplementary Material online) suggesting true positives in the genome-environmental analysis (Frachon et al. 2018, 2019). Six out of 33 of the environmental factors are significantly correlated with the genomic variance (PC1_genomic_) among populations (long-tongue bees, species strength, ratio C/N, fine silt, and Zn, supplementary table S6, Supplementary Material online). Using sensitive analyzes, it has been shown that the performance of GEA analyzes decreases when the correlation between environmental variable and first PC_genomic_ increase is strong (Frachon et al. 2018). To be conservative, the interpretation of the results for these six environmental variables deserves to be carefully considered, even though the correlations remain low (below 0.6). Second, we controlled for population structure in the genome-environmental association analyzes by using a covariance matrix of allele frequencies across populations implemented in the Bayesian hierarchical model used (Gautier 2015). Third, the obtained association scores were corrected by the local score approach, which proved to be efficient in reducing false positive rates in the genome-wide analyzes by considering surrounding genomic regions (Bonhomme et al. 2019).

**Fig. 3.**
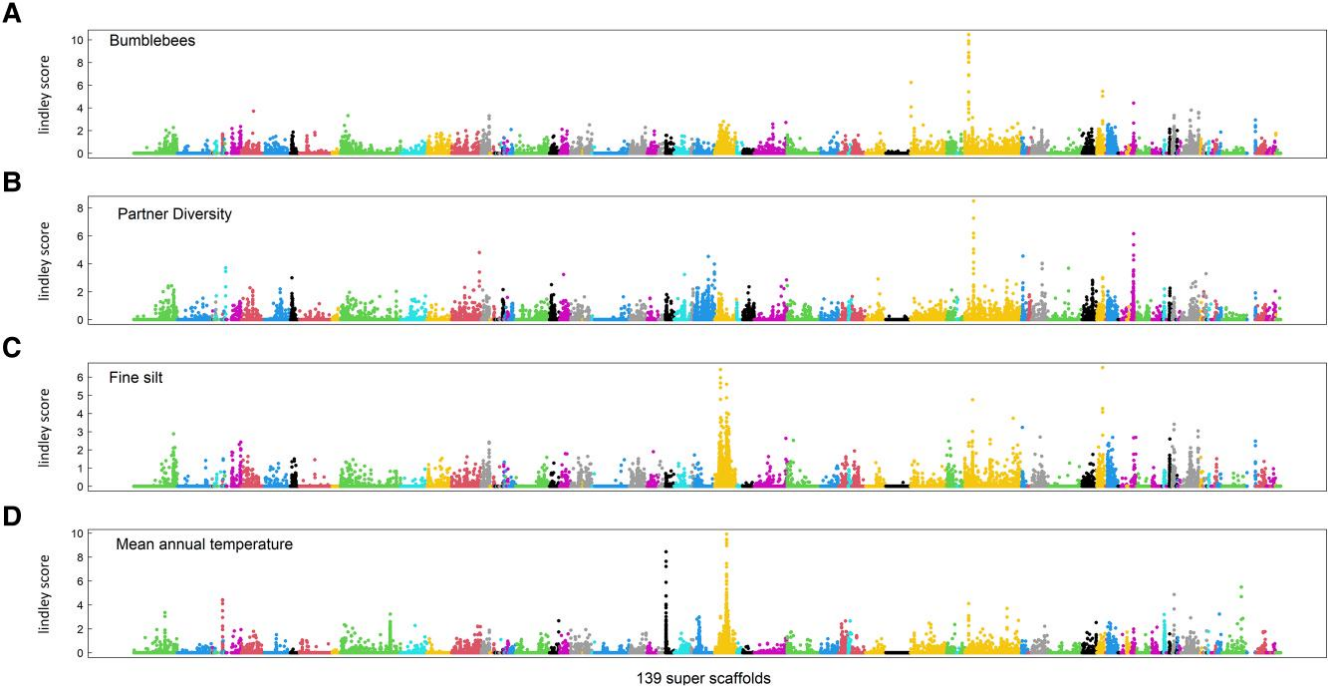
Manhattan plot of genome-environmental association analysis for four ecological variables; (*A*) visitation of bumblebees, (*B*) partner diversity, (*C*) fine silt, and (*D*) mean annual temperature. The *x-axis* indicates the physical position of the 5′530′708 SNPs along the 139 super-scaffolds illustrated by different colors. The *y-axis* indicates the Bayes factor corrected by the local score method (Lindley score).

To identify the putative adaptive genetic loci associated with visitation by specific functional categories of pollinators (e.g., visitation by long-tongue bees or bumblebees), pollinator community composition (based on *B. incana*—pollinator network indices such as number and diversity of pollinator groups), climate, soil composition, and texture variation, we performed a genome-wide scan for associations between standardized allele frequency variation along the 139 super-scaffolds of *B. incana* genome and these 33 ecological variables using a Bayesian hierarchical model. Combining the Bayesian hierarchical model and local score approaches, we observed neat and narrow peaks of association across the 139 super-scaffolds for the considered ecological variables (fig. 3, supplementary fig. S4, Supplementary Material online). Most of the identified genomic regions associated with visitation by specific functional categories of pollinators were unique, except for the visits of bumblebees, hoverflies, and long-tongue bees (supplementary Figs S5 and S6, Supplementary Material online). With unique genomic regions we mean SNPs associated with variation in a given ecological variable that is not shared with any other variable. For instance, 56% of the top SNPs (i.e., 56% of the 0.05% of SNPs with the highest association score) associated with long-tongue bees (1,435/2,541 SNPs) were unique, as were 95% of SNPs associated with large bees (2,414/2,541 SNPs), and 97% of SNPs associated with honeybees (2,471/2,541 SNPs, supplementary fig. S5, Supplementary Material online). However, only 15% of the top SNPs were unique for the bumblebee or hoverfly visits (supplementary fig. S5, Supplementary Material online). The latter shared 22% of their SNPs with the highest association score between them, and an important part of SNPs with the texture of the soil (fine silt and coarse sand, supplementary fig. S5, Supplementary Material online). Overall, 88% of the top SNPs were unique across the GEA performed on pollinator functional categories indicating an important part of SNPs associated with visitation by functional categories of pollinators (supplementary fig. S6, Supplementary Material online).

**Fig. 4.**
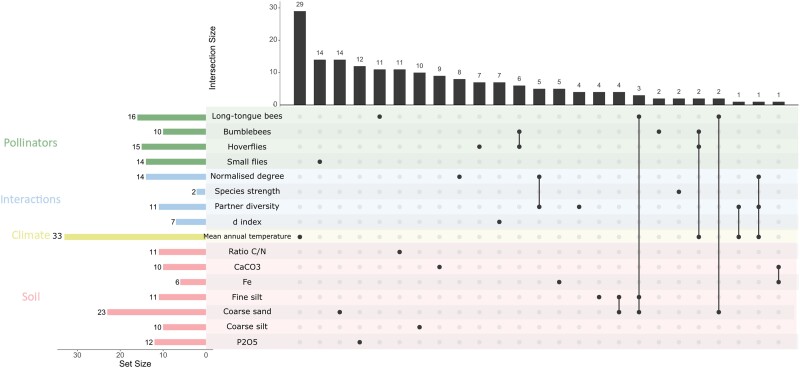
Illustration of the relationship among candidate genes associated with genomic local adaptation to ecological network. Only variables for which a significant enrichment of the selection signature was detected are considered. The left shows the number of candidate genes (set size) identified in genomic local adaptation to the specific variable in GEA analysis. On top, the number of candidate genes associated with a specific variable (single black dot) or shared among variables (multiple black dots linked). The candidate genes are those from the significant zones identified by correcting with the local score method the GEA, and the down and upstream genes.

Interestingly, the genomic architecture associated with pollinator community composition was slightly more complex, with the detection of multiple narrow peaks per variable (fig. 3, supplementary fig. S4, Supplementary Material online). As expected from their ecological correlations, some indices describing pollinator community composition shared genomic regions among them (fig. 4 and supplementary fig. S5, Supplementary Material online). Considering all the network indices related to pollinator communities, 73% of SNPs were associated with variation in these indices (supplementary fig. S6, Supplementary Material online). The remaining SNPs were shared among the indices and the variables associated with mean annual temperature, texture of the soil (fine silt and coarse sand), and visitation by some functional categories of pollinators (supplementary fig. S5, Supplementary Material online). Finally, 92% and 85% of SNPs with the highest association scores were uniquely associated with soil and climate, respectively (supplementary fig. S6, Supplementary Material online). Overall, our results highlighted a flexible genetic architecture involving mainly unique genomic regions associated with ecological variables, as well as few shared genomic regions among them.

### Putative Signals of Local Adaptation of *Brassica incana* to Combinations of Ecological Factors

To address the putative signatures of selection along the genome, we performed a genome-wide scan for the spatial genomic differentiation index X^T^X (an analogous to traditional F_ST_-based; Günther and Coop 2013) among the 21 natural populations of *B. incana* based on standardized allelic frequencies (i.e., allele frequencies corrected for population structure). After correcting the signal with the local score method (reducing potential false positives), we detected four genomic regions with strong spatial genomic differentiation among populations on super-scaffolds 1 (including five candidate genes), 10 (including three candidate genes), 37 (including nine candidate genes), and 74 (including 32 candidate genes, supplementary fig. S7, table S8, Supplementary Material online).

For each of 33 ecological variables, we tested for potential over-representation of SNPs with highest association scores (0.05% upper tail of BFdB distribution) in the extreme tail of the spatial genomic differentiation index (X^T^X) distribution (i.e., 0.05% of the most genomically differentiated SNPs among populations) as described in Brachi et al. (2015). The significance of these enrichments was estimated based on 10,000 null permutations as described in Hancock et al. (2011). These enrichments in signatures of selection allow us to distinguish the ecological variables for which genomic regions associated with their variation are potentially due to selective processes (significant enrichment) from those potentially due to neutrality processes (nonsignificant enrichment). We found that 17 out of 33 ecological variables displayed a significant enrichment (table 1, supplementary table S7, Supplementary Material online). For instance, we found a strong enrichment between the environment associations and the tail of X^T^X distribution for visitation by five functional categories of pollinators including bumblebees (23-fold), hoverflies (25-fold), and long-tongue bees (21-fold, table 1). The four pollinator community indices showed significant enrichment ranging from 6-fold for species strength to 31 for specialization (the *d-*index) (table 1). Finally, abiotic factors showed significant fold-enrichment for 7 out of 15 edaphic variables [ranging from 12 for calcium carbonate (CaCO_3_) to 62 for coarse sand], and a strong significant enrichment for mean annual temperature (109-fold, table 1, supplementary table S7, Supplementary Material online). These enrichments in genomic spatial differentiation indexes (X^T^X) combined with our GEA results suggest potential signals of local adaptation of *B. incana* to pollinator communities and abiotic factors encountered in these populations.

**Table 1.** Significant Enrichment in Signatures of Selection for Pollinator Categories, Plant–Pollinators Interaction Network Indices, Climatic, and Edaphic Variables Testing the Over-Representation of the 0.05% Upper Tail of the Lindley Score Distribution in the 0.05% Upper Tail of the Genome-Wide Spatial Differentiation (X^T^X) Distribution. See supplementary Table S7, Supplementary Material online for the Enrichment's Results of the 33 Ecological Variables.

Traits	ntops	Enrichment	*P-*value
Long-tongue bees	21	16.53	**
Bumblebees	23	18.11	***
Large bees	15	11.81	**
Hoverflies	25	19.68	***
Small flies	6	4.72	*
Normalized degree	19	14.96	**
Species strength	6	4.72	*
Partner diversity	20	15.74	***
*d-*index	31	24.40	***
Mean annual temperature	109	85.81	***
Ratio C/N	13	10.23	**
CaCO_3_	12	9.45	**
Fe	39	30.70	***
Fine silt	59	46.45	***
Large sand	62	48.81	**
Large silt	31	24.40	**
P_2_O_5_	15	11.81	**

The significance of the enrichment test obtained by performing 10,000 circular null permutations of the 0.05% top SNPs is indicated by the following *P*-values: *0.05<*P*-value <0.01, **0.01<*P*-value <0.001, ***P-value <0.001.

### Candidate Genes Involved in Putative Signals of Plant Adaptation to Pollinators Visitation

The candidate genes involved in putative signals of *B. incana* adaptation to the ecological network were identified by retrieving genes within significant zones identified by the local score approach on the GEA analyzes results, as well as down- and upstream genes as described in Libourel et al. (2021). From the GEA results showing significant enrichment in X^T^X index, we identified 48 candidate genes involved in plant adaptive responses to visitation by functional categories of pollinators, and 26 candidate genes involved in adaptive responses to pollinator community composition. The list of all candidate genes and underlying gene function, using the proteomic SwissProt database is available in supplementary table S8, Supplementary Material online.

For candidate genes involved in putative signals of plant adaptation to visitation by pollinator functional categories, we found some genes involved in plant signals and rewards. For instance, we identified a gene important for the synthesis of 2-*C*-methyl-*D*-erythritol 2,4-cyclodiphosphate synthase (*ISPF*), important for the biosynthesis of terpenoids (Tarkowská and Strnad 2018). We also identified a gene involved in the biosynthesis of the enzyme dihydropyrimidine dehydrogenase [NADP(+)] (*PYD1*), which is important for β-alanine biosynthesis (Wang et al. 2021), a compound present in nectar.

We found several candidate genes involved in plant architecture and growth (e.g., *NAC031, NUP98A*, and *PMEI10*), in reproduction processes (e.g., *EXPB5* and *IP5P12*), and in immunity and plant defense (*BSK7*). It is noteworthy that 38% of the identified candidate genes involved in plant adaptive responses to pollinators were associated with proteins with unknown function. Finally, a few candidate genes mentioned above were involved in both adaptive responses of *B. incana* to bumblebees and hoverflies such as UV-B induced protein, or PYD1 (fig. 4, supplementary table S8, Supplementary Material online).

For candidate genes involved in the putative signals of adaptation to pollinator community composition, we found some genes involved in plant architecture and growth such as transcription factor BEE2, protein TGD2, and ethylene-responsive transcription factor *ERF024*. Some of these candidate genes were involved in controlling pollen tube growth such as *LLG3,* or in flowering time such as *PCFS4*. However, 48% of the identified candidate genes involved in the putative signals of *B. incana* adaption to pollinator community composition are associated with proteins with unknown functions.

Finally, we observed multiple shared candidate genes in the putative signals of *B. incana* adaption to pollinators and climatic factors: between mean annual temperature and hoverfly and bumblebee visitation, pollinator diversity, and combined pollinator diversity and realized number of pollinator links, as well as shared candidate genes to pollinators and edaphic factors, for example, between long-tongue bees and coarse sand, and coarse sand and fine silt (fig. 4, supplementary table S8, Supplementary Material online).

## Discussion

While pollinators provide essential ecosystem services (Klein et al. 2007; Potts et al. 2010), whether and how plants with generalized pollination systems adapt to geographic variation in pollinator communities, and the underlying genetic basis of this adaptation is still poorly documented. Using an ecological genomics approach, our study unraveled the genomic bases of putative signals of plant adaptation to pollinator communities and potentially interacting abiotic factors.

### Putative Signals of Adaptation to Local Functional Categories of Pollinators

We observed a mosaic of pollinators among our 21 natural populations of *B. incana* and documented a genomic signature of putative signals of adaptation of this generalist-pollinated plant species to functional categories of pollinators. These results are in line with few studies emphasizing the importance of pollinators in driving the floral evolution not only in specialist but also in generalist plant species (Gòmez, Perfectti, et al. 2009; Bodbyl Roels and Kelly 2011; Sahli and Conner 2011; Gòmez et al. 2015; Sobral et al. 2015; Gervasi and Schiestl 2017; Schiestl et al. 2018; de Manincor et al. 2021). Our study uncovers the underlying genomic mechanisms of these putative signals of adaption: a genomic architecture involving different genomic regions strongly associated with visitation by functional categories of pollinators. In particular, we have identified pollinator category-specific candidate genes including some that are potentially involved in biosynthetic pathways of plant signals and rewards to attract pollinators. For instance, we identified two interesting candidate genes involved in *B. incana* adaptive responses to long-tongue bees; a candidate gene encoding for the enzyme *ISPF* involved in the ethylerythritol phosphate (*MEP*) pathway, responsible for terpenoids biosynthesis (mono- and diterpenoids biosynthesis; Abbas et al. 2017; Tarkowská and Strnad 2018; Bouwmeester et al. 2019), an important class of volatiles in plant–pollinator interactions (Baldwin et al. 2006; Abbas et al. 2017; Bouwmeester et al. 2019). In addition, we found a candidate gene encoding for the trehalose-phosphate phosphatase B (*TPPB*) enzyme involved in carbon flux maintenance correlated with sucrose supply (Nunes et al. 2013), an essential component of nectar. Interestingly, we identified genomic regions involved in putative signals of *B. incana* adaption both to efficient pollinators in terms of pollen transfer (long-tongue bees, bumblebees, and other large bees), as well as to supposedly less efficient pollinators (hoverflies and small flies). Genomic regions involved in local adaptation to supposedly “inefficient” pollinators may be surprising since they contribute less to plant reproductive success. A first explanation is an adaptative response of the plant to avoid those interactors by reducing/excluding visitation. Alternatively, in hoverfly dominated populations, limited pollen transfer could result in morphological changes to ensure reproduction by selfing such as reduced flower size, a decrease of herkogamy (physical distance between reproductive organs), and reduced volatile emissions (Gervasi and Schiestl 2017). However, although hoverflies are supposedly less efficient pollinators compared to large bees, hoverflies can still provide efficient pollination (Jauker and Wolters 2008; Q. Rusman unpublished) as well as protection against herbivores, as the larvae of many species are predators. Indeed, spatial and temporal variation in selective regimes by the local interactions (Gòmez, Perfectti, et al. 2009) can increase the importance of hoverflies for pollination when bees are scarce or absent (Jauker and Wolters 2008; Ohashi et al. 2021).

Surprisingly, in our study, it appears that putative signals of adaption to bumblebees and hoverflies involved similar genomic regions. For instance, we identified a candidate gene encoding for a dihydropyrimidine dehydrogenase (*PYD1*) enzyme involved in the biosynthesis of β-alanine (Wang et al 2021). β-alanine is a component present in nectar and potentially relevant for flower visitation behavior of bumblebees in *Gentiana lutea* (Rossi et al. 2014). It would be interesting to compare the rate of β-alanine production among our populations in relation to the ratio of bumblebee and hoverfly visitations to better understand this adaptive response at the phenotypic level. Finally, among the identified candidate genes involved in putative signals of adaption to visitation by different functional categories of pollinators, we also identified candidate genes involved in protein chaperones, plant growth, plant immunity, and a non-negligeable part of proteins with unknown function (∼33% of candidate genes). Thus, plant species with generalized pollination system show putative signatures of genomic adaption to their generalist pollinator community associated with candidate genes that are involved in plant–insect interactions.

### Putative Signals of Genomic Adaptation to Multiple Ecological Factors

We demonstrated genome-wide putative signatures of adaptation for multispecies assemblages of pollinators, that is, pollinator communities composition. This agrees with previous studies in evolutionary ecology showing adaptive responses to pollinator communities, that is, to the whole range of pollinators interacting with the plant (Gòmez, Abdelaziz, et al. 2009; Gòmez, Perfectti, et al. 2009; Sahli and Conner 2011; Lomáscola et al. 2019). Interestingly, the genomic architecture underlying the putative signals of *B. incana* adaption to the pollinator community composition was not the sum of genetic variants specific to functional categories of pollinators. In other words, the genetic variants involved in the putative signals of plant adaptation to the pollinator community are completely different from those involved in the putative signals of adaptation to unique pollinator functional categories, indicating nonadditive selection acting on *B. incana* as previously observed in plant–plant interactions (Baron et al. 2015; Libourel et al. 2021). This assumption agrees with a previous evolutionary study in *B. rapa* observing nonadditive selection for floral traits where phenotypic evolution mediated by the combination of two pollinator species was different from that mediated by either pollinator in isolation (Schiestl et al. 2018). A phenotypic characterization of our populations is still needed to better understand this evolutionary process. Nonadditive selection seems to be a common process in natural populations caused by indirect ecological effects. Such effects remain unpredictable in the study of pairwise selection, and difficult to study due to infinite number of ecological factors to be considered (Sahli and Conner 2011; Terhorst et al. 2015).

To estimate the potential indirect effects of abiotic factors on plant–pollinator interactions, we compared shared genetic variants among putative signals of adaption to abiotic and biotic factors. We observed only few shared candidate genes involved in the putative signals of *B. incana* adaptation to long-tongue bees, structure of the soil, and temperature. Long-tongue bees, mostly the genus *Anthophora* in our study, are ground-nesting bees. Variation in soil texture could have a significant impact on their occurrence in populations (Antoine and Forrest 2021). However, further ecological characterization is needed to control for indirect effects such as the local climate, composition of lower soil layers, microbiomes, herbivores, and natural enemies, or surrounding (flowering) plants. In addition, due to the geology of Southern Italy, we had a strong confounding effect between population structure (controlled by Bayesian models and local scores) and the type of soil (following a Northwest—Southeast axis), likely leading to a decrease of GEA power to detect true genomic bases involved in local adaptation. By illustrating the effect of complex ecological networks and abiotic factors on generalist plants through a complex genomic architecture, our results highlighted the importance of considering ecological variables, including biotic and abiotic factors, in the adaptative landscape of generalist species to better understand their impact on plant evolution (Carvalheiro et al 2021). With the current declines in insect diversity and its potential impact on flowering plant reproductive success, we stress the need to expand knowledge of the adaptive potential of plants to pollinator communities using a multidisciplinary approach from ecology to molecular biology to genomics.

## Materials and Methods

### Natural Populations of *Brassica incana*

We used the nonmodel plant species *Brassica incana* (fig. 1), an allogamous and self-incompatible perennial species growing on cliffs, and mainly distributed in Southern Italy (Landucci et al. 2014; Ciancaleoni et al. 2018). This wild species is a close relative of *Brassica oleracea* crop species (Landucci et al. 2014; El-Esawi 2017). From the data available in the literature and our own observations, we found 40 populations in Southern Italy. We used 21 natural populations with safe access (fig. 1, supplementary table S1, Supplementary Material online) for which at least 20 individuals were present in spring 2018. The populations grew on two distinct types of soil: six populations on tuff soil and 15 populations on limestone soil (fig. 1, supplementary table S1, Supplementary Material online). The populations were located from 2- to 767-month elevation (average = 278 months, supplementary table S1, Supplementary Material online), with an average distance of 61.78 km (median = 41.9 km, minimum = 1.25 km, maximum = 168.6 km).

### Ecological Characterization

We characterized the soil of the 21 natural populations of *B. incana* during spring of 2018 by collecting two soil samples per population from the ground surface (maximum depth ∼10 cm). The samples were sent to the Soil Analysis Laboratory of Arras (INRA, France, https://www6.hautsdefrance.inrae.fr/las). Twenty-one soil compounds were measured (**Dataset2**): aluminum (Al), carbon (C), ration carbon/nitrogen (ratio C/N), calcium (Ca), total CaCO_3_, clay (< 0 µm), total copper (Cu), iron (Fe), fine sand (0.05 mm–0.2 mm), coarse sand (0.2–2 mm), fine silt (2 µm–20 µm), coarse silt (20 µm–50 µm), potassium (K), magnesium (Mg), manganese (Mn), total nitrogen (N), sodium (Na), organic matter (om), phosphorus (P2O5), silicon (Si), and zinc (Zn). We followed the same method as described in Brachi et al. (2013), and all protocols are available at https://www6.hautsdefrance.inrae.fr/las/Prestations/Catalogue-analytique.

We retrieved 20 biologically meaningful climatic variables (**Dataset2**) for the 21 populations from ClimateEU database (v4.63 software, described in Hamann et al. 2013). Like Frachon et al. (2018), the average data across 2003–2013 were used for these 20 climatic data related to temperature and precipitation.

We characterized pollinator communities in spring 2018 (for 17 out of 21 populations) and spring 2019 (for 19 out 21 of populations) for a total of 19 biotic variables (**Dataset1, Dataset2**). To fully characterize the pollinator communities, one to four sessions (on average three sessions) of observations were conducted in spring 2018 and 2019 (one session in 2018, and three in 2019). We recorded pollinator visitation for one hour starting at 11.30 AM in each population, with observations of 10 min per plant. On average, five plants were observed per session (median = 5 plants, maximum = 11 plants, minimum = 1 plant). We assigned visitors to 13 functional categories: bumblebees (genus *Bombus*), long-tongue bees (genus *Anthophora*), other large bees (called large bees, mostly genus *Andrena*), small bees, honeybees (genus *Apis*), large wasps, small flies, large flies, hoverflies, small beetles, large beetles, butterflies (mostly genus *Pieris*), and wasps. Due to the scarce number of wasp visits (only one visit in CHIU population), it was discarded from the dataset, except for the plant–flower visitor network.

Because our study was population-centered, we estimated the best linear unbiased predictions (BLUP, **Dataset2**), that is, the average number of pollinator visits per population using a mixed model in the R Studio environment (package lme4, Bates et al. 2015).


$$Y_{i}=\mu_{\mathrm{trait}}+\mathrm{population}+\varepsilon_{i}$$

where Y_i_ is BLUP for visits by functional categories of pollinators, *µ*_trait_ the overall average of the trait (observed number of visits of functional categories of pollinators), population is considered as a random effect, and *ε*_i_ is the residual variance.

The plant–flower visitor network was constructed using the *bipartite* package (Dormann et al. 2008) based on the total number of visits within the populations from the 12 distinct functional categories of pollinators among the 21 natural populations of *B. incana*. Similarly to the species–species interaction networks, category-level indices for each population were calculated using *bipartite* (Dormann 2011). We calculated eight indices as described in Dormann (2011) and called latter *B.incana*—pollinator network indices or pollinator community composition indices: 1) normalized degree representing the number of partner species in relation to the potential number of partner species, 2) species strength representing the sum of dependencies of each species, aiming at quantifying a species' relevance across all its partners, 3) species specificity index representing the coefficient of variation of interactions, normalized to values between 0 (low variability suggesting low specificity) and 1 (high variability suggesting high specificity), 4) partner diversity representing the Shannon diversity index of the interactions of each species, 5) effective partners representing the logbase to the power of “partner diversity” interpreting as the effective number of partners, if each partner was equally common, 6) proportional similarity representing the specialization measured as dissimilarity between resource use and availability, 7) proportional generality representing the effective partners' divided by effective number of resources; this is the quantitative version of proportional resource use or normalized degree (i.e., the number of partner species in relation to the potential number of partner species), and 8) *d*-index representing the specialisation of each species based on its discrimination from random selection of partners.

We performed Spearman correlations for the 61 ecological variables (11 functional categories of pollinators, 8 network indices, 20 climatic variables, and 22 edaphic variables) using the R package Hmisc (Harrell 2021). We pruned the set of variables using the pairwise Spearman correlations among variables, and only variables with spearman's *ρ* < 0.8 were retained for the genomic analysis. In total, we kept 33 ecological variables: 11 functional categories of pollinators, 4 network indices, 3 climatic variables, and 15 edaphic variables. We performed a principal component analysis representing the distribution of 33 ecological variables among the 21 populations using the ade4 package in R (Dray and Dufour 2007).

### De novo Reference Genome

#### DNA Extraction

We chose one individual from the island of Capri population as reference genome (CAPR in fig. 1), a stable population between 1984 and 2012 with low gene flow with cultivated plants (Ciancaleoni et al. 2018). Seeds from the Capri population were collected in 2017, sown in a phytotron in the summer of 2018 (24 h light, 21 °C, 60% humidity, watered twice a day), and grown in an air-conditioned greenhouse at the University of Zürich (22.5 °C, 50–60% of humidity, additional light). Before DNA extraction, plants were kept in the dark for 2 days, which reduced the amounts of polysaccharides that interfere with the DNA extraction yield. We modified the high-molecular-weight genomic DNA (gDNA) extraction protocol from Mayjonade et al. (2016) as described in Russo et al. (2022). Briefly, this extraction was performed in 23 parallel tubes to increase the quantity of final DNA. The 23 DNA extracts were pooled together, and purified using carboxylated magnetic beads as explained in Mayjonade et al. (2016). We measured 146 ng/µL of total DNA concentration using a nanodrop (ratio A-260/A-280 = 1.85, ratio A-260/A-230 = 2.19) and 152 ng/µL with Qubit. The purified sample was sent to the Functional Genomic Center of Zürich (FGCZ) for library preparation and three different next-generation sequencing were performed to obtain a de novo reference genome of *Brassica incana*.

#### PacBio Library Preparation and Sequencing

The continuous long read SMRT bell library was produced using the single-molecule real-time (SMRT) bell Express Template Prep Kit 1.0. (Pacific Biosciences) at the FGCZ. The input gDNA concentration was measured using a Qubit Fluorometer dsDNA Broad Range assay (Thermo). The high-molecular weight gDNA sample (6 μg) was mechanically sheared to an average size distribution of 30 kbp, using a g-TUBE (Covaris) on a minispin plus centrifuge (Eppendorf). A Femto Pulse gDNA analysis assay (Agilent) was used to assess the fragment size distribution. Sheared gDNA was DNA damage repaired and end-repaired using polishing enzymes. PacBio sequencing adapters were ligated to the DNA template, according to the manufacturer's instructions. A Blue Pippin device (Sage Science) was used to size-select the SMRT bell library and enrich for fragments > 25 kbp. The size selected library was quality inspected and quantified using a Femto Pulse gDNA analysis assay (Agilent) and a Qubit Fluorimeter (Thermo), respectively. A ready-to-sequence SMRT bell-polymerase complex was created using the Sequel binding kit 3.0 (Pacific Biosciences P/N 101-500-400) according to the manufacturer’s instructions. The Pacific Biosciences Sequel instrument was programed to sequence the library on five Sequel™ SMRT^®^ cells 1 M v3 (Pacific Biosciences), taking one movie of 10 h per cell, using the Sequel Sequencing Kit 3.0 (Pacific Biosciences). After the run, the sequencing data quality was checked, via the PacBio SMRT Link software (v 6.0.0.47841), using the “run QC module” (supplementary table S3, Supplementary Material online).

#### Illumina Library Preparation and Sequencing

The TruSeq DNA Nano Sample Prep Kit v2 (Illumina, Inc., California, USA) was used in the succeeding steps. DNA samples (100 ng) were sonicated with the Covaris using settings specific to the fragment size of 350 bp. The fragmented DNA samples were size-selected using AMpure beads, end-repaired, and adenylated. TruSeq adapters containing unique dual indices for multiplexing were ligated to the size-selected DNA samples. Fragments containing TruSeq adapters on both ends were selectively enriched by polymerase chain reaction (PCR). The quality and quantity of the enriched libraries were validated using Tapestation (Agilent, Waldbronn, Germany). The product was a smear with an average fragment size of approximately 500 bp. The libraries were normalized to 10 nM in Tris–Cl 10 mM, pH 8.5 with 0.1% Tween 20. The Novaseq 6000 (Illumina, Inc., California, USA) was used for cluster generation and sequencing according to standard protocol. Sequencing was paired end (PE) at 2 X150 bp. This described protocol was used for both de novo sequencing of the reference individual, as well as the pool-sequencing of the 21 natural populations.

#### Preprocessing and Mapping of Illumina Reads

Quality control and Bowtie2 alignment of the Illumina PE reads were performed using data analysis workflows in the R-meta package ezRun (https://github.com/uzh/ezRun), managed by the data analysis framework SUSHI (Hatakeyama et al. 2016), which was developed and maintained by FGCZ. Technical quality was evaluated using FastQC (v0.11.7). We screened for possible contaminations using FastqScreen (v0.11.1) against a customized database in ezRun, which consists of SILVA rRNA sequences (https://www.arb-silva.de/), UniVec (https://www.ncbi.nlm.nih.gov/tools/vecscreen/univec/) sequences, refseq mRNA sequences and selected refseq genome sequences (human, mouse, *Arabidopsis*, bacteria, virus, phix, lambda, and mycoplasma) (https://www.ncbi.nlm.nih.gov/refseq/). Illumina PE reads were preprocessed using fastp (v0.20.0), with which sequencing adapters and low-quality ends (4 bp sliding windows from both ends, average quality < Q20) were trimmed. Trimmed reads passing the filtering criteria (average quality ≥ Q20, minimum length ≥ 18 bp) were aligned using Bowtie2 (v2.4.1) with the “–very-sensitive” option. Trimmed reads from the reference individual were aligned to the PacBio HG4P4 assembled contigs for genome polishing. Afterward, trimmed reads from the 21 natural populations were aligned to the polished and scaffolded genome assembly for variant analysis. PCR-duplicates were marked using Picard (v2.18.0). Read alignments were comprehensively evaluated using the mapping QC app in ezRun in terms of different aspects of DNA-seq experiments, such as sequence and mapping quality, sequencing depth, coverage uniformity, and read distribution over the genome (supplementary table S3, Supplementary Material online).

#### De novo Genome Assembly

PacBio subreads from all five SMRT cells were merged and assembled using HGAP4 (Hierarchical Genome Assembly Process v4) in the PacBio SMRT Link software (v 6.0.0.47841). Before being assembled, subreads were filtered with reading quality of 70%. The estimated genome size was set at 650 Mbp. Illumina PE reads from the same sample were preprocessed and mapped to the assembled primary contigs as described above. Assembled primary contig sequences were then further polished with mapped Illumina PE reads using pilon (v1.23). Only reads with mapping quality above Q20 and bases with phred scores above Q20 were used for the polishing.

#### In silico Genome Digestion and Bionano Optical Mapping

The polished genome assembly was first in silico digested using Bionano Access software (v1.2.1) to evaluate whether the nicking enzyme (Nb.BspQI), with recognition sequence GCTCTTC, and the non-nicking enzyme DLE-1, with recognition sequence CTTAAG, were suitable for optical mapping in the genome. An average of 13.6 nicks/100 kbp with a nick-to-nick distance N50 of 13,734 bp was expected for Nb.BspQI, while DLE-1 was found to induce 22.2 nicks/100 kbp with a nick-to-nick distance N50 of 8,054 bp. The values were in line with manufacturer’s requirements.

For the direct label and stain (DLS) protocol, the DNA sample was labeled using the Bionano Prep DNA labeling kit-DLS (cat. no. 80005) according to manufacturer’s instructions. In detail, 750 ng of purified gDNA was labeled with DLE-1 labeling mix and subsequently incubated with proteinase K (Qiagen, cat. no. 158920) followed by drop dialysis. After the clean-up step, the DNA was prestained, homogenized, and quantified using a Qubit Fluorometer to establish the appropriate amount of backbone stain. The reaction was incubated at room temperature for at least 2 h. For the nick label repair and stain (NLRS) protocol, the DNA sample was labeled using the Bionano Prep DNA labeling kit-NLRS according to the manufacturer’s instructions (Bionano Genomics, cat. no. 80001). In detail, 300 ng of purified gDNA was nicked with Nb.BspQI (New England BioLabs, cat. no. R0644S) in NEB Buffer 3. The nicked DNA was labeled with a fluorescent- deoxyuridine triphosphate (fluorescent-dUTP) nucleotide analog using Taq DNA polymerase (New England BioLabs, cat. no. M0267S). After labeling, nicks were ligated with Taq DNA ligase (New England BioLabs, cat. no. M0208S) in the presence of deoxynucleotide triphosphates (dNTPs). The backbone of fluorescently labeled DNA was counterstained overnight with YOYO-1 (Bionano Genomics, cat. no. 80001). DLS and NLRS labeled DNA samples were loaded into a nanochannel array of a Saphyr Chip (Bionano Genomics, cat. no. FC-030-01) and run by electrophoresis each into a compartment. Linearized DNA molecules were imaged using the Saphyr system and associated software (Bionano Genomics, cat. no. 90001 and CR-002-01). BioNano row molecule data are available in supplementary table S3, Supplementary Material online.

#### Assembly of Optical Maps and Hybrid Scaffolding

The de novo assembly of the optical maps was performed using the Bionano Access (v1.2.1) and Bionano Solve (v3.2.1) software. The assembly type performed was the “Saphyr data”, “nonhuman”, “nonhaplotype” with “extend and split” and “cut segdups”. Default parameters were adjusted to accommodate the genomic properties of the *Brassica incana* genome. Specifically, the “initial *P-*value” cutoff threshold was adjusted to 1 × 10^−10^ and the *P-*value cutoff threshold for extension and refinement was set to 1 × 10^−11^ according to manufacturer’s guidelines (default values are 1 × 10^−11^ and 1 × 10^−12^, respectively). Dual-enzyme hybrid scaffolding was then performed using the same software suits with default parameters. This dual-enzyme hybrid scaffolding used the Bionano optical maps to scaffold polished (PacBio and Illumina) contigs.

#### Genome Annotation

Repeat sequences in the de novo assembled genome were predicted using RepeatScount (v1.0.6). Predicted repeat sequences and known transposable elements in *Brassica oleracea* were masked using RepeatMasker (v4.1). Gene model prediction was performed using maker (v3.01.03). In detail, ab initio gene prediction was performed using AUGUSTUS with the pretrained parameter set for *Arabidopsis*. Protein and cDNA sequences of *B. oleracea* (ensemble release 42) were aligned to the assembled genome and used as supporting evidence for gene prediction. For functional annotation, prediction protein sequences were compared to the SwissProt database (release 2019_03) using blastp (v2.6.0), and the InterPro database using interproscan (v5.32–71.0). For BLASTP comparison against SWISSProt we used an *E*-value cutoff of 1 × 10^−6^. The best hit, if existed above the *E*-value cutoff, was used to annotate the corresponding *Brassica incana* genes.

### Genomic Characterization of 21 Populations Using a Pool-Sequencing Approach

In spring 2018, we collected leave tissue from, on average, 28 individuals per population (median = 30 plants, max = 30 plants, min = 15 plants, i.e., a total of 590 samples) in 1.5 mL Eppendorf tubes. The samples were stored during the field day in dry ice and moved into a −80 °C freezer at the end of field day. The DNA extraction was performed in the fall of 2018 by grinding samples using two beads, cooling them down in liquid nitrogen, and crushing them with 30 vibrations/second three times 30 s. We extracted DNA using the sbeadex maxi plant kit from LGC Genomics in Kingfisher Flex Purification Systems (Thermo Scientific), a magnetic-particle robot at the genetic diversity center Zürich platform. We added 250 µL of lysis buffer in all homogenized samples. After homogenization (2–3 s on vortex, and 20 reversing tubes), we incubated our samples for 20 min at 65 °C. We added 1.12 µL of RNAse (940 U/mL) and reversed tubes ten times. We incubated the samples for ten more minutes at 65 °C. After centrifuging at 2.5 × 1,000 rcf for 10 min at 20 °C, we transferred 200 µL of the lysate in deep 96-well plates with 520 µL of binding buffer and 60 µL of sbeadex particles suspension. The samples were incorporated into the Kingfisher robot for the DNA purification. After bringing magnets into contact with the tubes for 1 min, the supernatant was removed and discarded. 400 µL of wash buffer PN1 was added to each sample and mixed by pipetting to resuspend the pellet. After 10 min of incubation and agitation at room temperature, the magnets were brought into contact with the tubes for 1 min. The supernatant was removed and discarded, and a second round of washing was performed adding 400 µL of wash buffer PN2 in each sample, incubating 10 min at room temperature, and bringing the magnets into contact with tubes. The supernatant was removed and discarded. 100 µL of elution buffer PN was added to the pellet and mixed by pipetting. The solution was incubated at 55 °C for 10 min, and finally, the magnets were brought into contact with tubes for 3 min until the sbeadex formed a pellet and stayed on the magnets. The eluate of the samples was transfered to a new 96-well plate and stored in the fridge.

The DNA concentration of all samples was measured using ddDNA Qubit assay measurement on plate reader Spark M10 (excitation wavelength = 485 nm, emission wavelength = 535 nm). Eight samples with too low DNA concentration were discarded. In total 582 samples were used for the pooled sequencing with a DNA concentration superior to 1.5 ng/µL (average = 12.68 ng/µL, median = 10.18 ng/µL, max = 61.09 ng/µL). For each of the 21 populations, the individuals were pooled together equimolarly, with an average of 27.7 individual per pool (median = 29 individuals, minimum = 15 individuals, maximum = 30 individuals). We proceeded with the pool-sequencing as previously described in the methods for the de novo reference genome sequencing using Illumina sequencing.

#### Freebayes Variant Calling

Multi-samples frequency-based (-F 0.05) variant calls (−use-best-n-alleles four-pooled-continuous) were generated using the freebayes-parallel script in freebayes (v1.2.0-4-gd15209e, Garrison and Marth 2012), with 16 threads of freebayes running in parallel across regions of 100 kb in the de novo polished genome assembly (PacBio, Illumina, and Bionano). SNPs with variant quality above Q20 were retained using bcftool (v1.9) for downstream analysis and were annotated with de novo predicted gene models using SnpEff (v4.2). The final dataset was composed of 6,899,774 SNPs across the 21 natural populations of *B. incana*.

### Data Filtering

The matrix of population allele frequencies was trimmed using VCFtools (Danecek et al. 2011) and following Frachon et al. (2018). We kept only biallelic loci (391,671 SNPs discarded) and removed the indels (7,960 SNPs discarded). We discarded SNPs with a minimum mean read depth lower than six, and higher than 100 (143,710 SNPs discarded). We removed all SNPs with missing values in more than two populations (613,387 SNPs discarded). We finally kept only 139 super-scaffolds (203,955 SNPs discarded). The final allele read count matrix included 5,530,708 SNPs for 21 populations.

### Genome-Environment Association Analysis on 33 Ecological Variables

We performed a GEA analysis using a pool-sequencing approach between the 5,530,708 SNPs and 15 variables describing pollinator communities (11 functional categories of pollinators, and four *B. incana*—pollinator network indices), three climatic variables, and 15 edaphic variables. Genome scans were based on a Bayesian hierarchical model implemented in Baypass software (Gautier 2015). Considering the covariance matrix of allele frequencies among populations, this model allowed to correct potential effect of demographic histories (Gautier 2015). As described in Frachon et al. (2019), we used the core model to estimate the Bayesian factor (BF_is_ in dB called later BFdB) between the allelic frequencies along the genome, and different descriptors of pollinator communities as well as abiotic variables. The core model was repeated three times due to the importance of sampling algorithms, and the final Bayesian Factor was estimated by averaging the three models. Considering the large amounts of SNPs used, we subsampled the procedure to estimate the matrix of population allele frequencies (Ω) as in Frachon et al. (2018), by dividing the full data set into 19 subdatasets of ∼ 254,785 SNPs each. The GEA for each trait and each subdata set were performed in parallel and merged again after analyzes. Finally, we corrected the BFdB obtained by using a local score approach to consider the linkage disequilibrium (Bonhomme et al. 2019). This allows for the detection of the accumulation of similar *P-values* in the same region increasing the power of genomic analyzes. To do this, we artificially created *P-values* by ranking the BFdB values from highest to smallest and divided the rank by the total number of SNPs. The parameter *ξ* was fixed at three for the local score method (Bonhomme et al. 2019; Libourel et al. 2021). We used upset plots to detect shared SNPs and candidate genes among the 33 ecological variables considering 0.05% SNPs with highest association score after applying the local score method (R package UpSetR, Gehlenborg et al. 2019). Due to the geology of Southern Italy, we observed a Northwest–Southeast axis of variation for type of soil (tuff vs. limestone), potentially matching the demographic history. We estimated the genomic variation among the population using a SVD of the matrix of raw allele frequency (without population structure correction). A strong significant correlation between the genomic variation from SVD and environmental variables could lead to a decrease in the power of the GEA analysis.

### Putative Signatures of Selection

We performed a genome-wide scan of the spatial genomic differentiation index (X^T^X) among the 21 populations (Günther and Coop 2013; Gautier 2015). This index considered the standardized allele frequencies of a given SNP, a measure of the variance of allele frequencies across 21 natural populations. This method has been demonstrated to be successful for natural populations (Frachon et al. 2018, 2019). As described above, we also implemented the local score approach to correct the X^T^X fixing parameter *ξ* = 3 (Fariello et al. 2017; Bonhomme et al. 2019). Finally, we estimated the enrichment in spatial genomic differentiation index (X^T^X) by testing whether the SNPs with the highest association scores with environmental variables (0.05% upper tail of the BFdB corrected by local score method) were significantly enriched in the 0.05% extreme upper tail of X^T^X distribution (Brachi et al. 2015; Frachon et al. 2018, 2019). The significance of the enrichment was tested using the method described by Hancock et al. (2011) by running 10,000 null circular permutations of the 0.05% SNPs with the highest association score with 33 environmental variables.

### Identification of Candidate Genes

To identify candidate genes involved in putative signals of *B. incana* adaptation to pollinator communities and abiotic variables, we retrieved genes within the significant zone identified by the GEA analyzes and corrected by the local score approach, and down and upstream genes of these zones as in Libourel et al. (2021). Only significant zones containing more than three SNPs were kept. The functions of the genes were predicted using the SwissProt database (release 2019_03).

## Supplementary Material

msad036_Supplementary_Data

## Data Availability

All data are available as described hereafter. Sequencing data from Pacbio and Illumina used for this study are available at the ENA database (accession number PRJEB54646). The bionano raw data and assembled optical maps are available at NCBI database (sample name PRJNA859008). All scripts and datasets are available at Dryad database (doi:10.5061/dryad.pnvx0k6r0).
