# Supplementary material for: Putative Signals of Generalist Plant Species Adaptation to Local Pollinator Communities and Abiotic Factors
Source: Mol Biol Evol. 2023 Feb 16;40(3):msad036. doi: 10.1093/molbev/msad036 (PMC10015620; doi:10.1093/molbev/msad036)
Supplement: msad036_Supplementary_Data [file msad036_supplementary_data.zip › SI_Frachon_et_al_MBE.pdf]

# Supplementary information

**Title:** Genomic adaptation of a generalist plant species to local pollinator communities and abiotic factors

**Authors:** Frachon L., Arrigo L., Rusman Q., Poveda L., Qi W., Scopece G., Schiestl P.F.

**Figure S1. Matrix of spearman correlations of 61 ecological variables.** Significant correlations are indicated by coloured dots. No dot means no significant correlation. The direction of the coloration is indicated by the blue and red gradient (gradient scale at the lower part of the figure). The traits indicated in grey were discarded from the genomic analysis due to high correlations with other traits (spearman *rho* > 0.8).

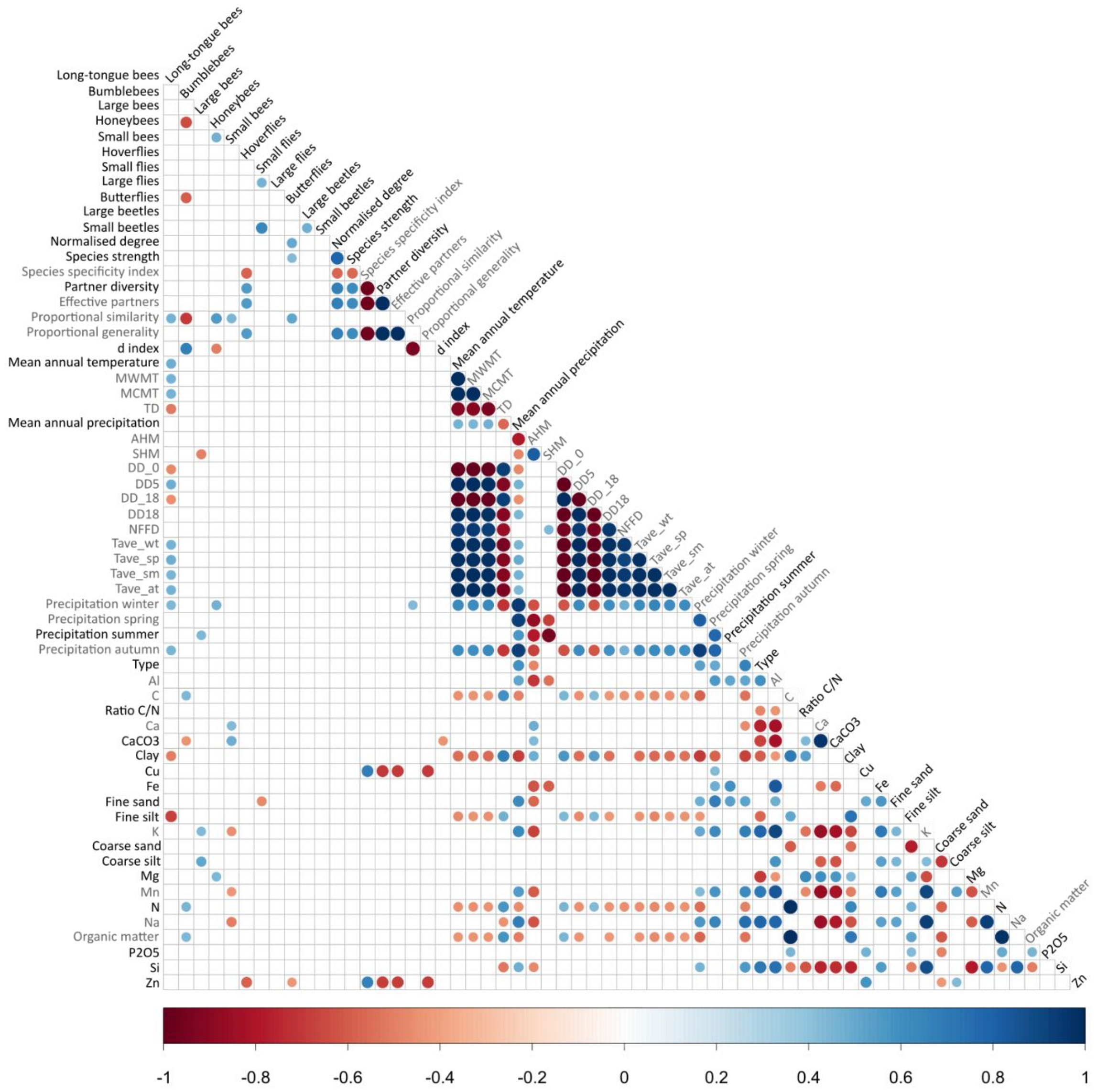

**Figure S2. Ecological variation among the 21 natural populations of *B. incana*. (A)** Correlation plot from a principal component analysis preformed on 33 environmental variables with pairwise rho spearman < 0.8. Principal components 1 and 2 explained 21.62% and 17.13% respectively. **(B)** Position of the 21 natural populations of *B. incana* in ecological space. The populations in tuff soil are coloured in orange, and in limestone soil in blue.

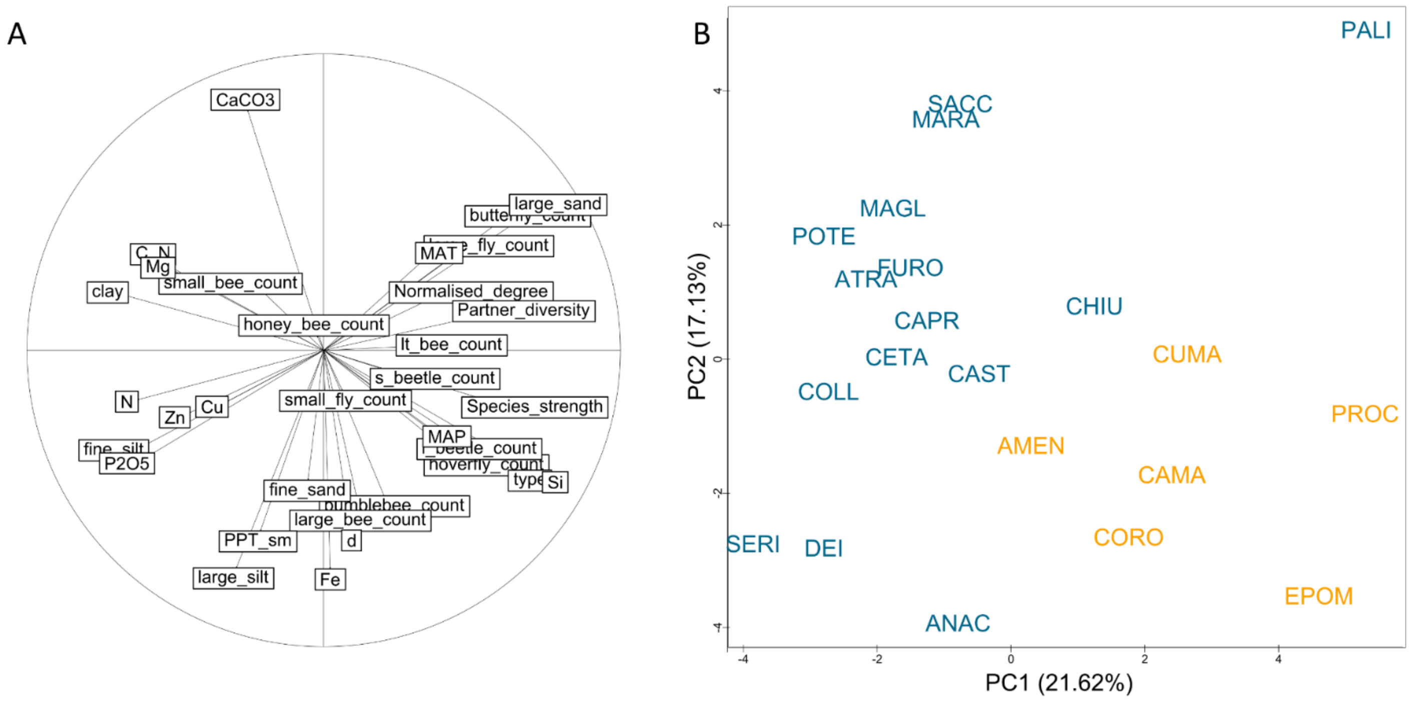

**Figure S3. Position of the 21 natural populations of *B. incana* in genomic space.** Genomic variation was estimated using a singular value decomposition (SVD) of omega matrix using Baypass software from one sub-sample. The first principal component (PC) explains 94.3% of the genomic variance, and the second PC explains 3% of the genomic variance. The tuff and limestone soils of the 21 populations are indicated in orange and blue, respectively.

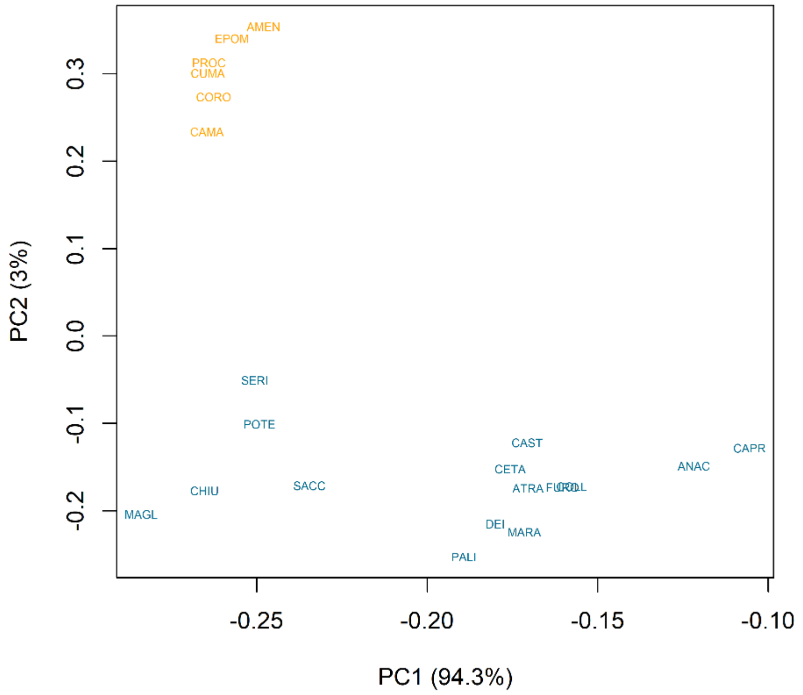

**Figure S4. Manhattan plots of Genome-Environmental Association performed on 33 ecological variables.** The x-axis represents the physical position of SNPs along the 139 super-scaffolds illustrated in colour. The *y-axis* is the Lindley score. The name of the ecological variable is indicated on the upper part of the Manhattan plot.

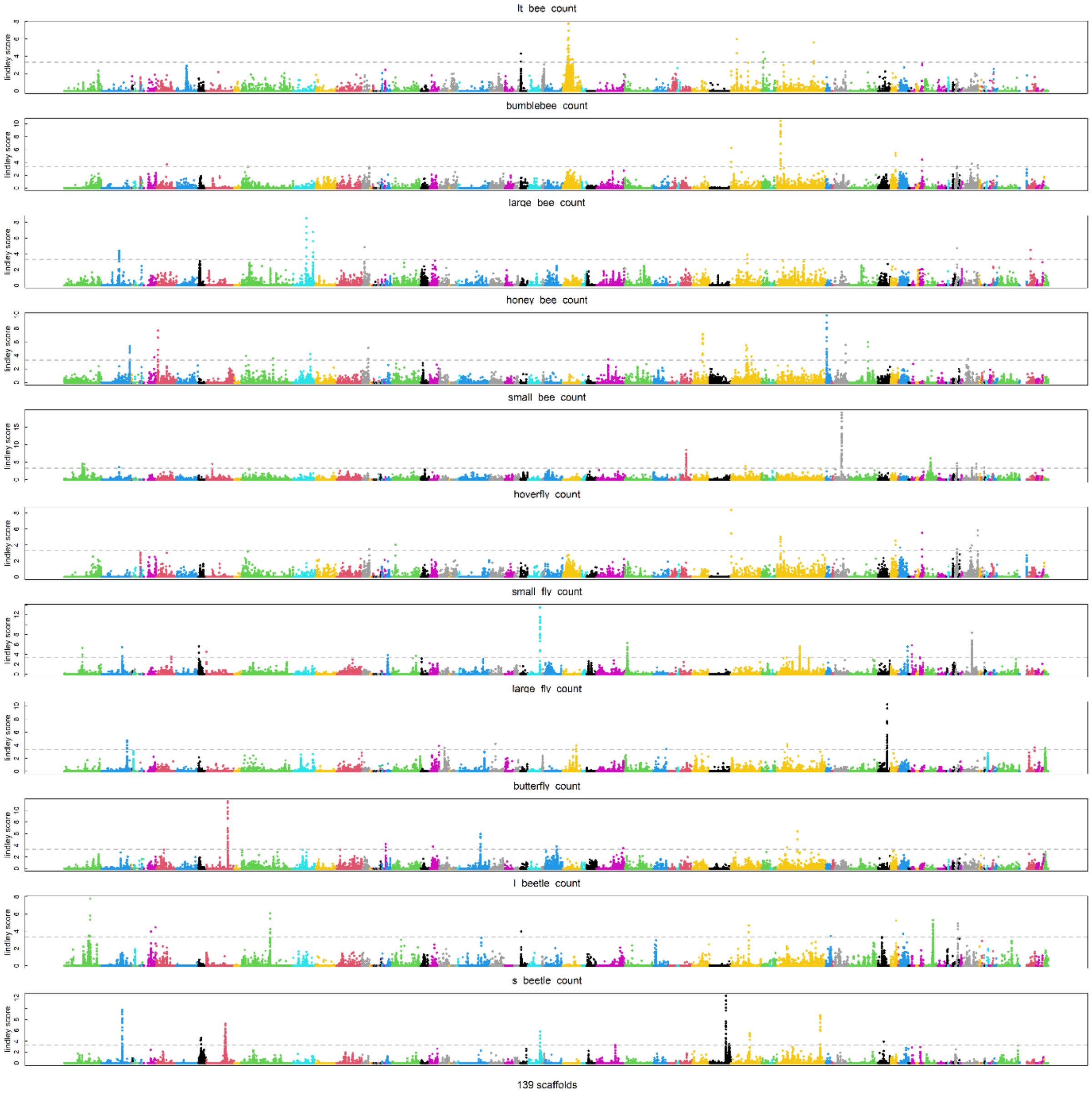

**Figure S4. To be continued**

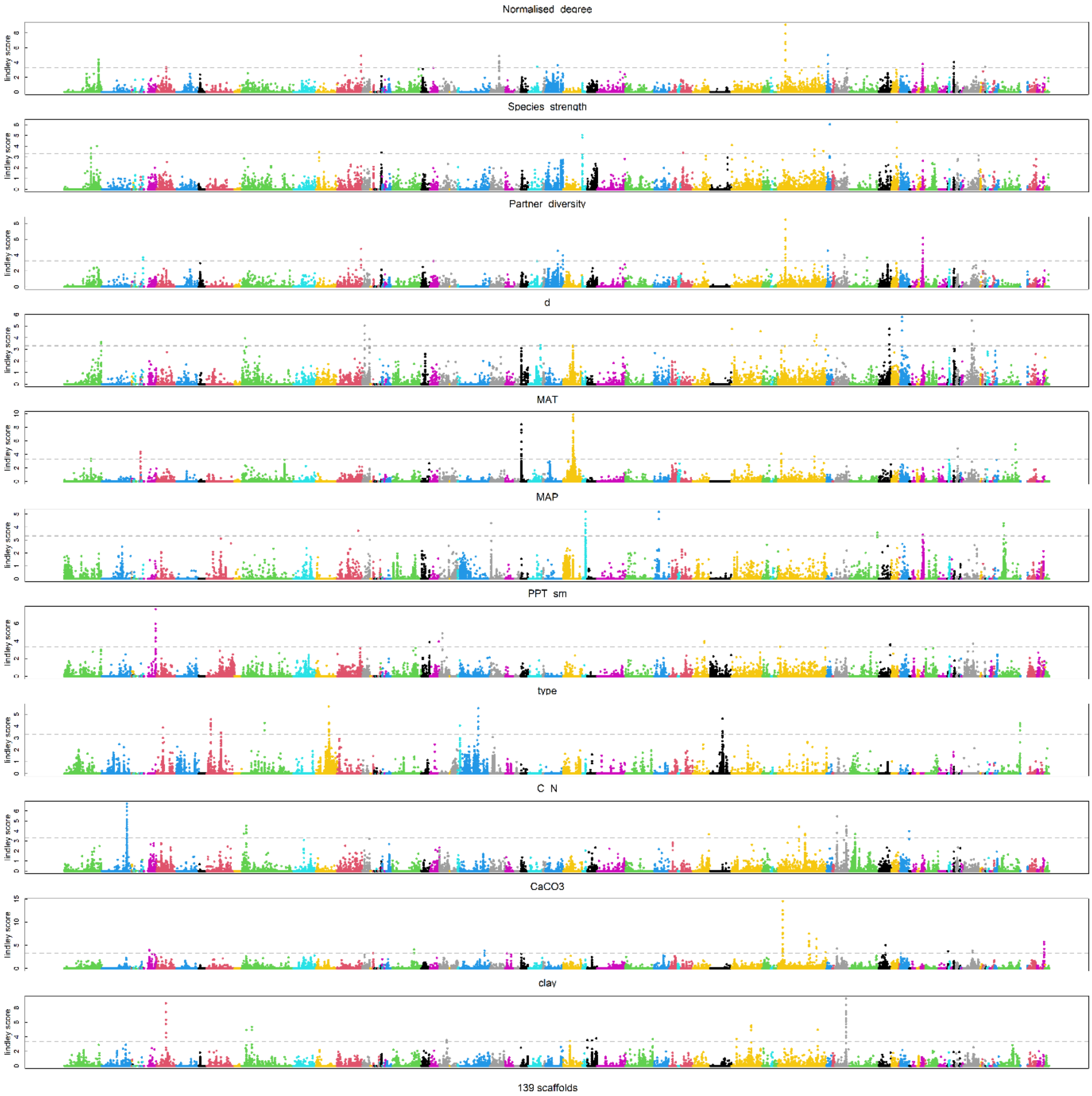

## Figure S4. To be continued

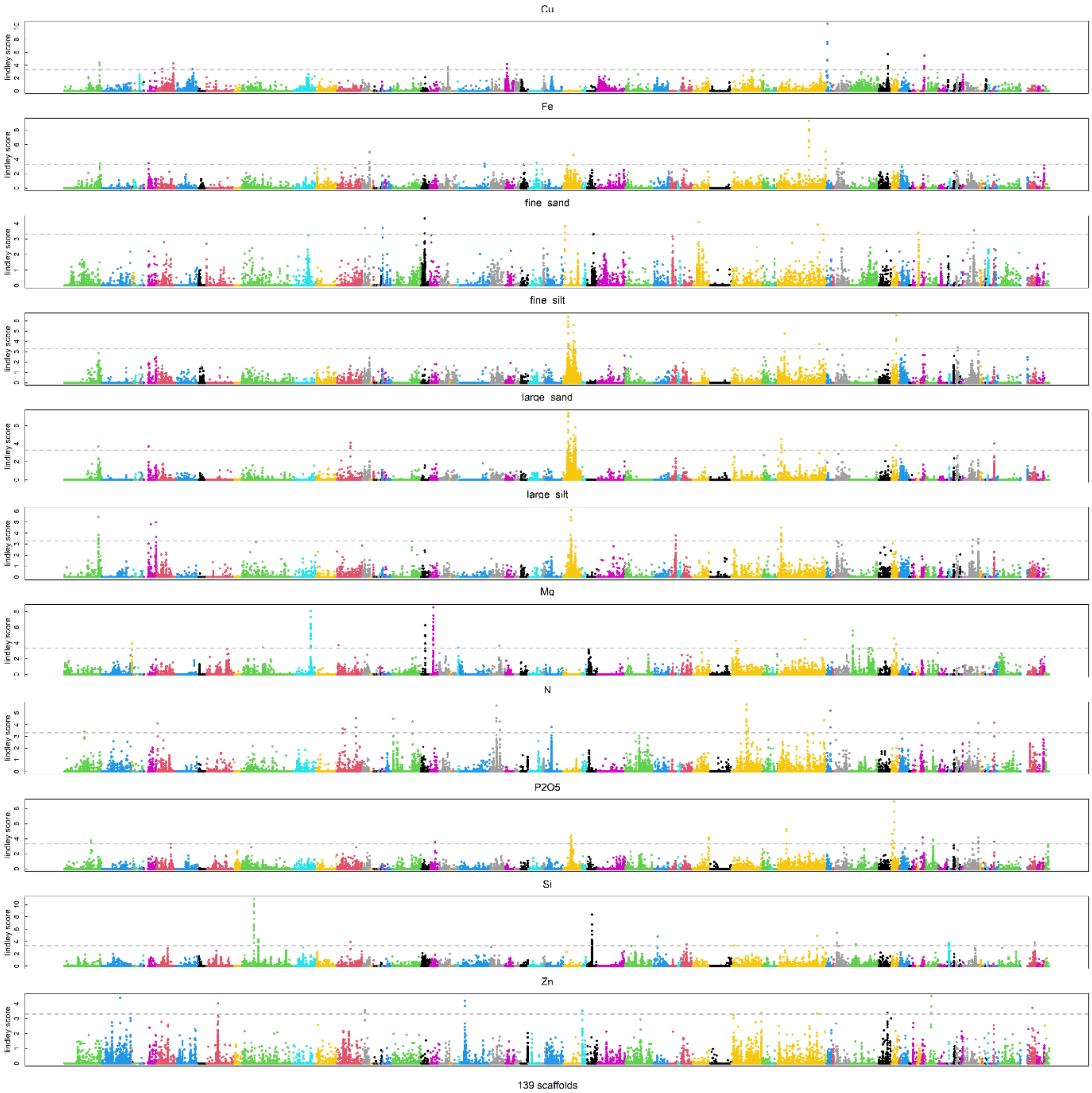

**Figure S5. Illustration of the variability of SNPs involved in plant adaptation to a complex ecological network and abiotic factors.** The upset plot illustrates the specific SNPs for 33 ecological variables (only dots), and the shared SNPs among these ecological variables (dots linked with bar). The blue bars represent SNPs shared among *B. incana* responses to pollinators community composition and visitation by functional categories of pollinators. The yellow bars represent the SNPs shared among the climate variables and pollinators (functional categories and network indices). The red bars represent the SNPs shared among edaphic variables and the pollinators. The top 0.05% SNPs of the highest association score were considered for 33 ecological variables listed in the left (*i.e*. set size = 2541 SNPs per ecological variable). Only the 156 first intercepts are shown (*i.e*. more than 1% of the set size). For instance, 1435 SNPs are unique to long-tongue bees, and 149 SNPs are shared between long-tongue bees and mean annual temperature. The ecological variables with non-significant enrichment in signature of selection have been shaded.

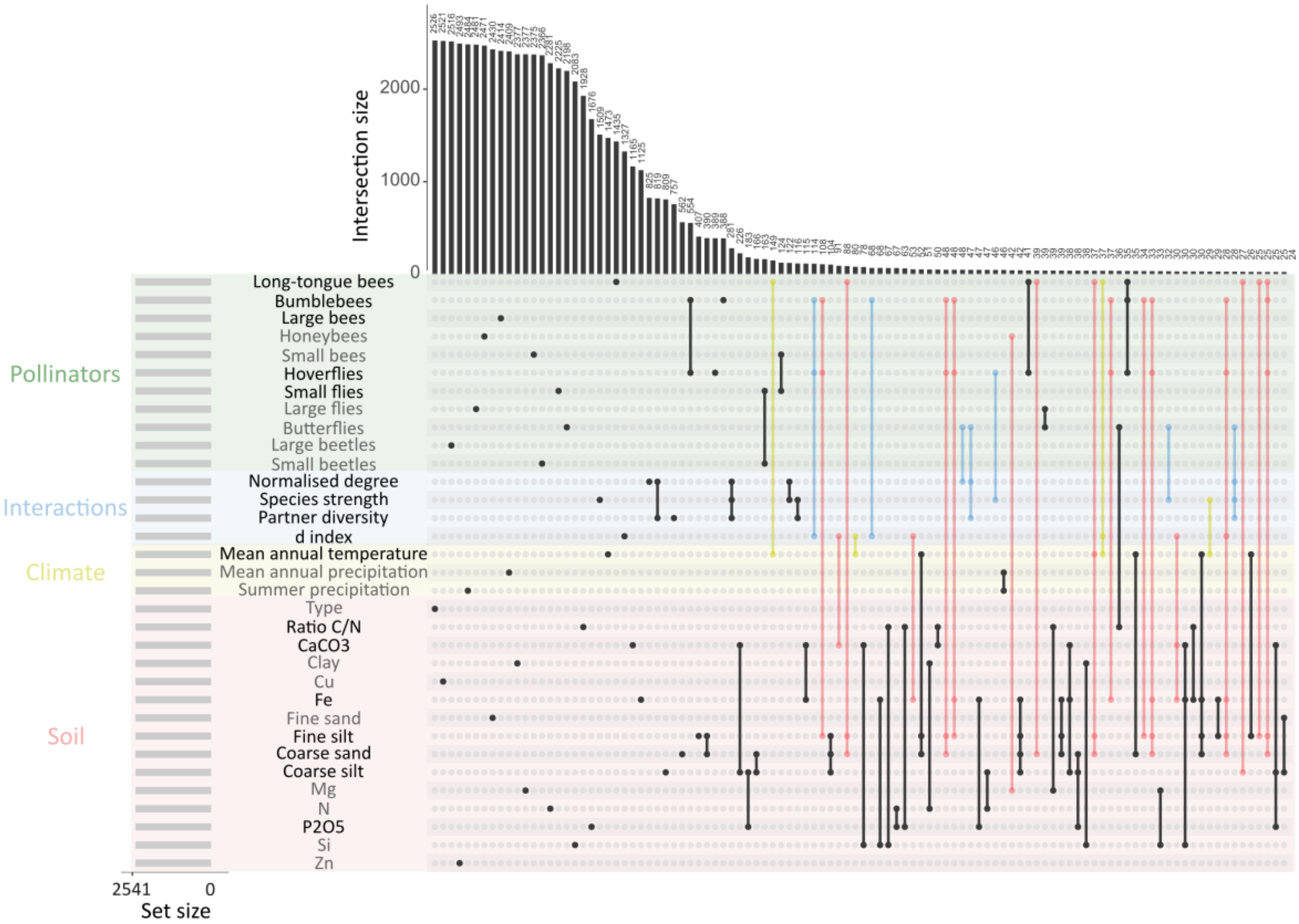

**Figure S6: Illustration of the flexibility of the genetic architecture of *Brassica incana* in response to a complex ecological network and abiotic factors.** Venn diagram illustrating the shared top SNPs (0.05% of the highest local score) for the 33 ecological variables. The variables are grouped by main categories (pollinator categories in green, pollinators community composition in blue, edaphic variables in red, and climatic variables in yellow). The number of variables and the total number of SNPs considered are indicated between parenthesis bellow each category of variables. The Venn diagram was draw using jvenn.toulouse.inra.fr website.

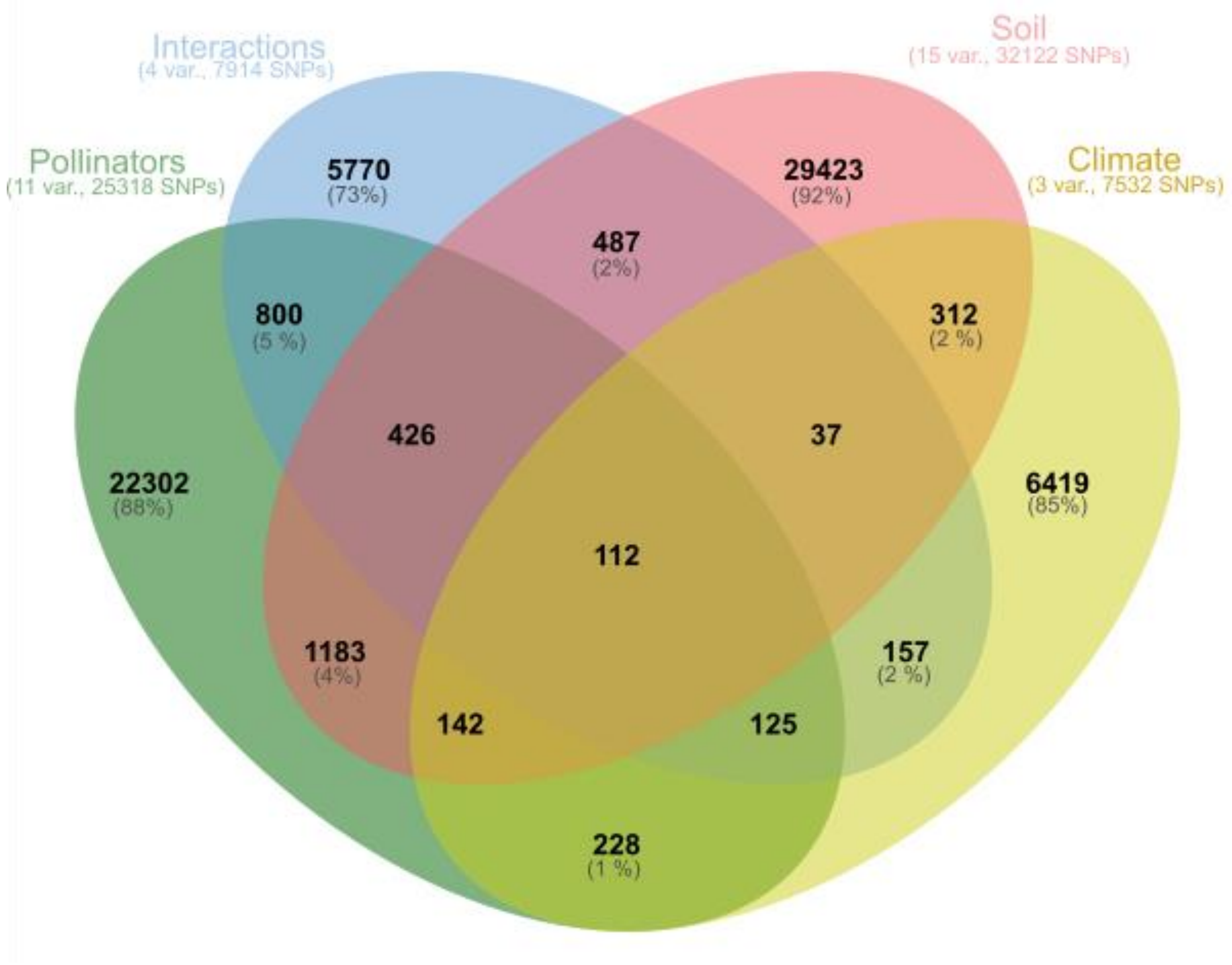

**Figure S7. Manhattan plot of the index of genetic differentiation genetic (XtX).** The upper panel illustrated the results obtained by Baybass analysis, and the lower panel those obtained by correcting with the local score method (*y-axis* is the Lindley score). The *x-axis* represents the physical regions of the SNPs along the 139 super-scaffolds.

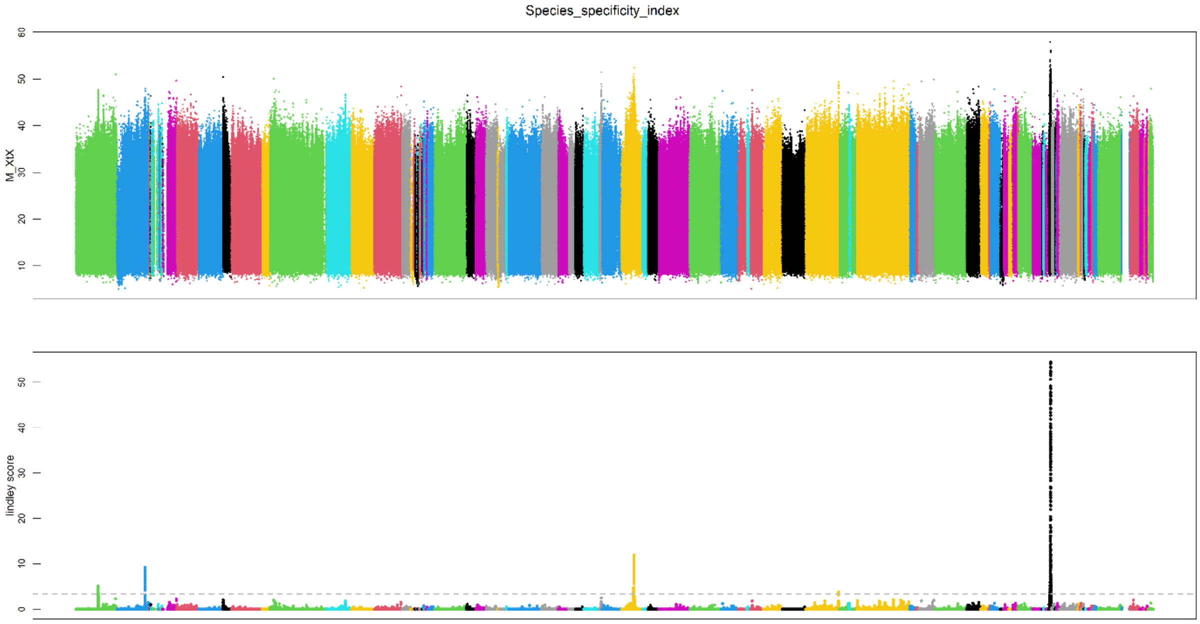

**Table S1. Description of the 21 natural populations of *Brassica incana*.**

| Pop. Name | Localities | latitude | longitude | Elevation | Substrate | Pop. Size 2018 |
|---|---|---|---|---|---|---|
| PROC | Monte di Procida | 40.809370 | 14.044790 | 16 | Tuff | 20 |
| AMEN | Ischia, Lacco Ameno | 40.751632 | 13.896623 | 6 | Tuff | 30 |
| EPOM | Ischia, Vetta Epomeo | 40.730072 | 13.895328 | 767 | Tuff | 20 |
| CUMA | Mainland di Cuma | 40.850475 | 14.049944 | 41 | Tuff | 20 |
| CORO | Napoli, Coroglio-Nisida | 40.798367 | 14.175846 | 19 | Tuff | 40 |
| CAPR | Capri, Scala Fenicia | 40.556357 | 14.228237 | 180 | limestone | 100 |
| ANAC | Anacapri, Monte Solaro | 40.545771 | 14.223282 | 562 | limestone | 1000 |
| CHIU | Valico di Chiunzi | 40.719061 | 14.619117 | 648 | limestone | 15-30 |
| COLL | Colli-Positano | 40.619627 | 14.447449 | 253 | limestone | 15-30 |
| FURO | Furore | 40.614468 | 14.548246 | 91 | limestone | 15-2 |
| CAMA | Napoli, Camaldoli | 40,855181 | 14.207769 | 159 | Tuff | 20-30 |
| MAGL | Magliano Vetere | 40.342298 | 15.242826 | 622 | limestone | 15-20 |
| PALI | Palinuro, Arco naturale | 40.030830 | 15.308058 | 2 | limestone | 30-50 |
| ATRA | Atrani | 40.636737 | 14.610037 | 52 | limestone | 15 |
| SERI | Serino | 40.8204531 | 14.919611 | 708 | limestone | 10-15 |
| SACC | Sacco, Salerne | 40.386551 | 15.366420 | 505 | limestone | 50-100 |
| CAST | Castellammare di Stabia | 40.682200 | 14.439804 | 20 | limestone | 50 |
| DEI | Sentiero degli Dei | 40.625781 | 14.536430 | 649 | limestone | 15 |
| CETA | Cetara | 40.645236 | 14.699832 | 52 | limestone | 30 |
| POTE | Vietri di potenza | 40.570945 | 15.520259 | 359 | limestone | 30 |
| MARA | Maratea | 40.0421848 | 15.6525735 | 135 | limestone | 100 |

**Table S2. Indices from the *Brassica incana* -- pollinator network analysis.** The description of the indices is available in the methods and in Dormann 2011. In grey, the indices discarded for the genomic analysis due to high correlation with other ecological factors (See Figure S1).

| Populations | Normalised degree | Species strength | Species specificity index | Partner diversity | Effective partners | Proportional similarity | Proportional generality | d |
|---|---|---|---|---|---|---|---|---|
| AMEN | 0.42 | 0.18 | 0.73 | 0.85 | 2.35 | 0.43 | 0.38 | 0.21 |
| ANAC | 0.67 | 0.88 | 0.44 | 1.56 | 4.76 | 0.38 | 0.77 | 0.39 |
| ATRA | 0.67 | 0.44 | 0.58 | 1.23 | 3.41 | 0.66 | 0.55 | 0.13 |
| CAMA | 0.50 | 0.40 | 0.46 | 1.47 | 4.35 | 0.62 | 0.70 | 0.14 |
| CAPR | 0.25 | 0.09 | 0.79 | 0.63 | 1.88 | 0.44 | 0.30 | 0.20 |
| CAST | 0.58 | 0.27 | 0.53 | 1.34 | 3.80 | 0.76 | 0.61 | 0.07 |
| CETA | 0.75 | 0.68 | 0.66 | 1.19 | 3.28 | 0.51 | 0.53 | 0.20 |
| CHIU | 0.67 | 1.55 | 0.48 | 1.46 | 4.29 | 0.72 | 0.69 | 0.10 |
| COLL | 0.25 | 0.03 | 0.77 | 0.68 | 1.98 | 0.38 | 0.32 | 0.17 |
| CORO | 0.50 | 0.58 | 0.51 | 1.31 | 3.70 | 0.72 | 0.60 | 0.10 |
| CUMA | 0.58 | 0.56 | 0.54 | 1.32 | 3.74 | 0.74 | 0.60 | 0.09 |
| DEI | 0.17 | 0.04 | 0.72 | 0.64 | 1.89 | 0.14 | 0.31 | 0.32 |
| EPOM | 0.67 | 1.71 | 0.37 | 1.74 | 5.67 | 0.26 | 0.92 | 0.53 |
| FURO | 0.67 | 0.21 | 0.44 | 1.58 | 4.84 | 0.80 | 0.78 | 0.04 |
| MAGL | 0.67 | 0.67 | 0.47 | 1.48 | 4.41 | 0.78 | 0.71 | 0.06 |
| MARA | 0.50 | 0.42 | 0.53 | 1.26 | 3.52 | 0.65 | 0.57 | 0.12 |
| PALI | 0.67 | 0.67 | 0.42 | 1.61 | 5.02 | 0.66 | 0.81 | 0.14 |
| POTE | 0.50 | 0.10 | 0.46 | 1.43 | 4.19 | 0.69 | 0.68 | 0.09 |
| PROC | 0.83 | 1.49 | 0.39 | 1.80 | 6.06 | 0.70 | 0.98 | 0.09 |
| SACC | 0.67 | 0.48 | 0.55 | 1.33 | 3.77 | 0.70 | 0.61 | 0.11 |
| SERI | 0.58 | 0.58 | 0.69 | 1.08 | 2.95 | 0.48 | 0.48 | 0.23 |

**Table S3. Sequence data collected for *de novo* genome assembly of *Brassica incana* from Pacbio and Illumina.**

| | PacBio CLR | Illumina PE reads |
|---|---|---|
| Number of reads | 2,481,304 | 249,786,052 |
| Number of bases (bp) | 47,372,198,992 | 74,935,815,600 |
| Read N50 | 17 kbp | 2X150 bp |
| Estimated coverage* | 73 X | 115 X |

*assumed genome size of 650 Mbp

**Table S4. Bionano row molecule data collected for hybrid scaffolding of *Brassica incana* contigs.**

<table>
<tr><td colspan="2">Protocol</td><td>NLRS</td><td>DLS</td></tr>
<tr><td colspan="2">Enzyme</td><td>Nb.BspQI</td><td>DLE-1</td></tr>
<tr><td rowspan="2">Molecule >= 20 kbp</td><td>Total length (Mbp)</td><td>1,694</td><td>2,278</td></tr>
<tr><td>N50 (Mbp)</td><td>0.101</td><td>0.123</td></tr>
<tr><td rowspan="3">Molecule >= 150 kbp</td><td>Total length (Mbp)</td><td>549</td><td>972</td></tr>
<tr><td>N50 (Mbp)</td><td>233</td><td>0.267</td></tr>
<tr><td>Label density</td><td>5.43/100 kbp</td><td>5.03/100 kbp</td></tr>
<tr><td>Effective coverage</td><td></td><td>340.88</td><td>34.82</td></tr>
</table>

**Table S5. Final assembly statistics of *Brassica incana* contigs and scaffolds.**

| Metrics | Contigs | Scaffolds | Un-anchored contigs |
|---|---|---|---|
| Number of sequences | 1,339 | 139 | 824 |
| Total sequence length (Mbp) | 664 | 617 | 73 |
| Sequence N50 (Mbp) | 1.53 | 12 | 0.16 |
| Longest sequence (Mbp) | 12 | 32 | 1 |

**Table S6. Spearman correlations between genomic variance (SVG), 33 ecological variables, and PC1 and PC2 from the principal analysis performed on 33 ecological variables.**

| | PC1 (94.3%) | |
|---|---|---|
| | rho | *P* |
| long tongue bees | -0.44 | * |
| bumblebees | 0.18 | *ns* |
| large bees | -0.43 | *ns* |
| honeybees | -0.15 | *ns* |
| small bees | -0.26 | *ns* |
| hoverflies | -0.13 | *ns* |
| small flies | -0.13 | *ns* |
| large flies | -0.22 | *ns* |
| butterflies | -0.08 | *ns* |
| large beetles | 0.09 | *ns* |
| small beetles | 0.25 | *ns* |
| Normalised degree | -0.19 | *ns* |
| Species strength | -0.47 | * |
| Partner diversity | -0.37 | *ns* |
| d | 0.29 | *ns* |
| Mean annual temperature | 0.25 | *ns* |
| Mean annual precipitation | -0.12 | *ns* |
| Summer precipitation | -0.11 | *ns* |
| type | -0.54 | * |
| Ratio C/N | 0.53 | * |
| CaCO3 | 0.21 | *ns* |
| clay | 0.28 | *ns* |
| Cu | 0.31 | *ns* |
| Fe | 0.12 | *ns* |
| Fine sand | 0.29 | *ns* |
| Fine silt | 0.48 | * |
| Coarse sand | -0.33 | *ns* |
| Coarse silt | -0.08 | *ns* |
| Mg | 0.33 | *ns* |
| N | 0.07 | *ns* |
| P2O5 | 0.07 | *ns* |
| Si | -0.23 | *ns* |
| Zn | 0.44 | * |
| Ecological PC1 | -0.39 | *ns* |
| Ecological PC2 | 0.04 | *ns* |

**Table S7.** Enrichment in signature of selection for 33 ecological variables including pollinator categories (11 variables), pollinator community composition indices (4 variables), climatic (3 variables) and edaphic variables (15 variables) in the 0.05% upper tail of the Lindley score distribution in the 0.05% upper tail of the genome-wide spatial differentiation (XtX) distribution.

| Traits | ntops | Enrichment | pvalue |
|---|---|---|---|
| Long-tongue bees | 21 | 16.53 | ** |
| Bumblebees | 23 | 18.11 | *** |
| Large bees | 15 | 11.81 | ** |
| Honeybees | 1 | 0.79 | *ns* |
| Small bees | 1 | 0.79 | *ns* |
| Hoverflies | 25 | 19.68 | *** |
| Small flies | 6 | 4.72 | * |
| Large flies | 0 | 0.00 | *ns* |
| Butterflies | 2 | 1.57 | *ns* |
| Large beetles | 0 | 0.00 | *ns* |
| Small beetles | 0 | 0.00 | *ns* |
| Normalised degree | 19 | 14.96 | ** |
| Species strength | 6 | 4.72 | * |
| Partner diversity | 20 | 15.74 | *** |
| d index | 31 | 24.40 | *** |
| Mean annual temperature | 109 | 85.81 | *** |
| Mean annual precipitation | 4 | 3.15 | *ns* |
| Summer precipitation | 0 | 0.00 | *ns* |
| type | 0 | 0.00 | *ns* |
| Ratio C/N | 13 | 10.23 | ** |
| CaCO3 | 12 | 9.45 | ** |
| clay | 0 | 0.00 | *ns* |
| Cu | 0 | 0.00 | *ns* |
| Fe | 39 | 30.70 | *** |
| Fine sand | 3 | 2.36 | *ns* |
| Fine silt | 59 | 46.45 | *** |
| Coarse sand | 62 | 48.81 | ** |
| Coarse silt | 31 | 24.40 | ** |
| Mg | 1 | 0.79 | *ns* |
| N | 3 | 2.36 | *ns* |
| P2O5 | 15 | 11.81 | ** |
| Si | 0 | 0.00 | *ns* |
| Zn | 3 | 2.36 | *ns* |

**Table S8.** Candidate genes are available in a separated file.
